# Isoimperatorin suppresses triple-negative breast cancer by modulating miR-874-3p/POU2F1 axis: a new avenue for metabolic and redox intervention

**DOI:** 10.1186/s40659-026-00678-x

**Published:** 2026-03-12

**Authors:** Shuo Wu, Yuexuan Su, Ruishan Zhang, Yuying Wang, Xiang Li

**Affiliations:** 1 Department of Blood Diseases and Breast Medicine, Cancer Hospital of China Medical University, Shenyang, China; 2 Cancer Hospital of Dalian University of Technology, Dalian, China; 3 Liaoning Cancer Hospital and Institute, Shenyang, China; 4 Department of Breast Surgery, Cancer Hospital of China Medical University, Shenyang, China

**Keywords:** Isoimperatorin, ROS, Breast cancer, Apoptosis, Glycolysis, Mitochondria, miR-874-3p, POU2F1

## Abstract

**Background:**

Metabolic alterations are key factors driving the development of breast cancer. Among them, metabolic reprogramming is an important biological hallmark of breast cancer. It is crucial to gain in-depth understanding of the relevant mechanisms and find effective treatment strategies. Isoimperatorin (ISO), a natural furanocoumarin, exhibits various pharmacological effects such as anti-inflammatory and antiviral activities. However, the impact of ISO on the growth of breast cancer cells and its underlying mechanisms remain unclear.

**Methods:**

Cell culture, transfection, and animal experiments were employed. CCK -8, colony formation, and flow cytometry were used to detect cell proliferation, apoptosis, and ROS levels. miRNA-Seq, qRT-PCR, and Western Blotting were applied to analyze gene and protein expression.

**Results:**

ISO inhibited breast cancer cell proliferation, induced apoptosis and ROS accumulation, and suppressed the Warburg effect. ISO upregulated miR- 874-3p, which directly targeted the 3′-UTR of POU2F1, inhibiting the transcriptional activation of key glycolytic genes. Blocking this axis reversed ISO’s tumor-suppressive effects in vitro and in vivo.

**Conclusion:**

This study first reveals a novel mechanism by which ISO regulates breast cancer metabolism and apoptosis via the miR-874-3p/POU2F1 axis, highlighting ISO’s potential as a dual metabolic-redox intervention agent and providing a new combined therapeutic target for breast cancer.

**Graphical abstract:**

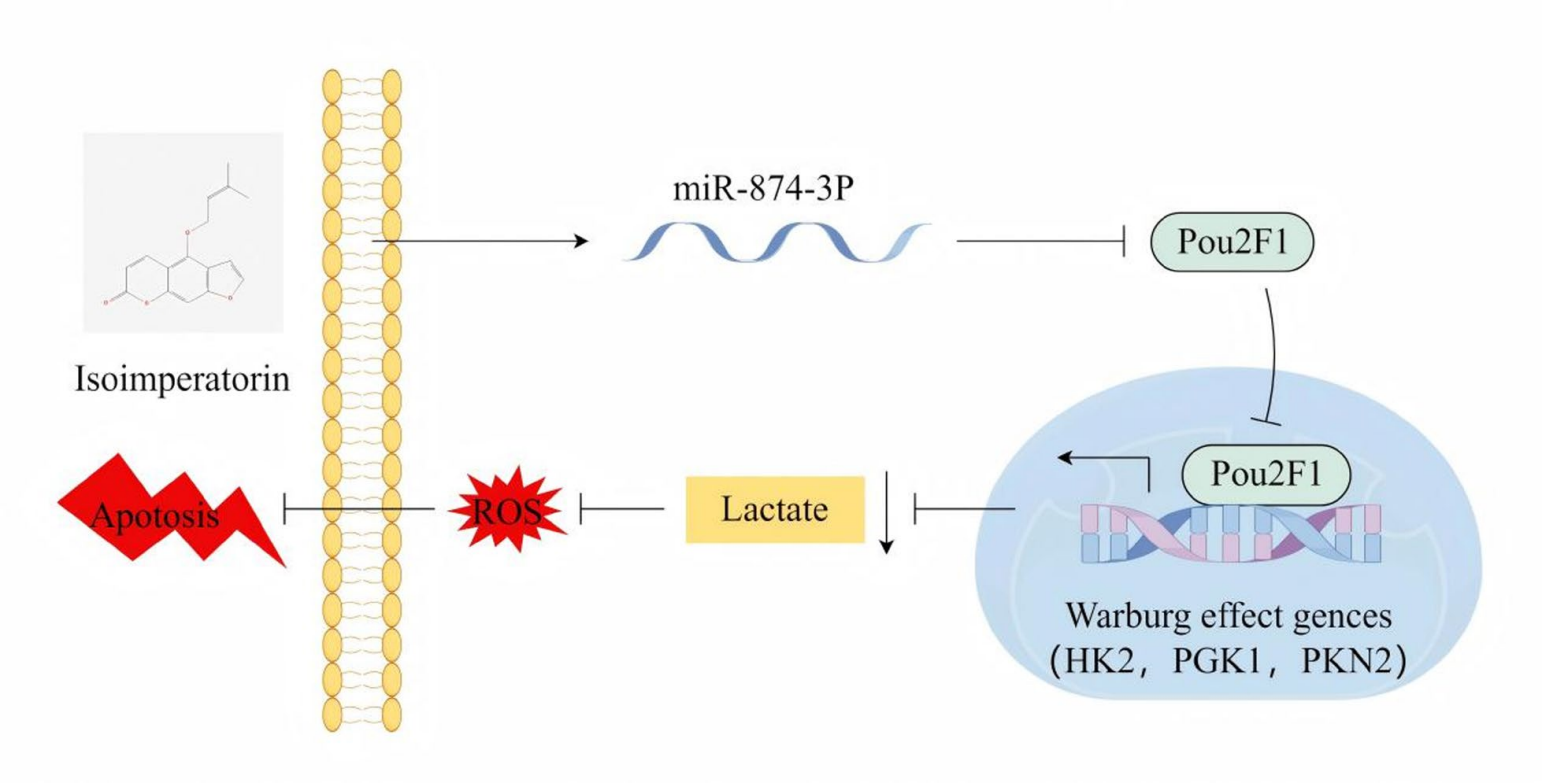

**Supplementary Information:**

The online version contains supplementary material available at 10.1186/s40659-026-00678-x.

## Introduction

Breast cancer, a malignant tumor of mammary epithelial tissue, has rising incidence and is the leading cause of cancer-related deaths in women [1]. The etiology of breast cancer is complex, involving not only hereditary factors but also hormonal metabolic disorders, stress, and negative emotions as high-risk contributors [2]. Advances in medical treatments, including surgical resection, chemotherapy, and localized radiotherapy, have significantly improved survival rates. However, patients’ lives are still at danger because to metastasis, recurrence, and toxicities linked to treatment [3, 4]. As a result, effective therapy and early diagnosis have attracted more attention recently.

Metabolic rewiring is a key biological hallmark of breast cancer [5]. This metabolic shift significantly influences the malignancy and poor prognosis of the disease. Despite the availability of sufficient oxygen, cancer cells preferentially engage in glycolysis, a phenomenon known as the Warburg effect or aerobic glycolysis. This process enables them to maintain elevated levels of glycolytic intermediates to meet biosynthetic demands while simultaneously decreasing the reactive oxygen species (ROS) produced through oxidative phosphorylation (OXPHOS) [6]. This adaptation enables their survival and rapid proliferation in nutrient-deprived tumor microenvironments. Moreover, glycolysis is closely linked to cellular redox balance. Glycolytic intermediates can also enter other metabolic pathways, such as the pentose phosphate pathway (PPP), to generate NADPH, which is critical for maintaining intracellular reduced glutathione (GSH) levels, thereby aiding ROS clearance and preserving redox homeostasis [7]. ROS has dual roles, exerting either beneficial or detrimental effects depending on its concentration. At moderate levels, ROS serves as a secondary messenger regulating various cellular functions, including the cell cycle, apoptosis, and gene expression. However, chronically high ROS levels can induce DNA damage, protein denaturation, and lipid peroxidation, ultimately leading to cell death. One study demonstrated that glycolytic inhibition significantly increased intracellular ROS levels in tumor cells, accompanied by upregulated apoptotic markers and reduced cell viability [8]. Similarly, another study found that suppressing glycolysis enhanced tumor cell sensitivity to chemotherapeutic drugs, partly due to elevated ROS levels amplifying drug-induced cell death [9]. Thus, targeting glycolysis and ROS balance represents a novel therapeutic focus in cancer management.

Natural products play a vital role in cancer drug discovery, with plant-derived chemotherapeutic agents forming part of current standard treatments [10]. Isoimperatorin (ISO), a natural furanocoumarin, exhibits diverse pharmacological effects, including anti-inflammatory [11], antiviral [12], anti-injury [13], and antitumor properties [14, 15]. Previous studies have shown ISO’s significant antitumor activity against the gastric cancer SGC-7901 cell line [16, 17]. ISO exhibits antiproliferative activity by acting as an apoptosis inducer through mitochondrial-mediated molecules such as Survivin, Bcl-2, Bax, and caspase-3/9, while also arresting the cell cycle at the G2/M phase [16, 17]. Additionally, ISO suppresses epithelial-mesenchymal transition by modulating the NF-κB signaling pathway and CXCR4 expression, exhibiting potent inhibitory effects in colorectal and liver cancers [14]. Furthermore, the herbal compound GuiErBai, which includes ISO, shows promising therapeutic potential against cervical cancer [18]. These findings lay the groundwork for exploring ISO as a potential anticancer agent. A structure-based multitarget molecular docking analysis revealed high affinity between ISO and breast cancer [19], but its mechanism in breast cancer is not fully understood.

## Materials and methods

### Cell culture

The human breast cancer cell line MDA-MB-231 and human breast epithelial cell line MCF-10 A, MCF-12 A were purchased from the Cell Bank of the Chinese Academy of Sciences (Shanghai, China). All cells were cultured in DMEM supplemented with 10% fetal bovine serum (Gibco, USA), 100 units/mL penicillin, and 100 µg/mL streptomycin (Life Science, USA). Cells were maintained in a humidified incubator at 37 °C with 5% CO_2_.

### Cell treatment and transfection

ISO (dissolved in dimethyl sulfoxide [DMSO], purchased from MedChemExpress, China) was used to incubate cells at the indicated concentrations. The miR-874-3p inhibitor, mimic, sh-POU2F1, and POU2F1 overexpression vector were purchased from RiboBio (Guangzhou, China). Cells were cultured in 6-well plates and transfected using Lipofectamine 3000 (Invitrogen, Carlsbad, CA, USA; catalog #L3000008).

### Animals

Female BALB/c nude mice (6–8 weeks old) were provided by Vital River (Beijing, China).Mice were housed in standard polypropylene cages with sterile corncob bedding under controlled temperature, humidity, and lighting (12-hour light/dark cycle). All animal experiments were approved by the Welfare and Ethical Committee for Experimental Animal Care of China Medical University (Protocol #XYZ).

### Tumor growth assay

Breast cancer cells (2 × 10^6^ cells per nude mouse) were subcutaneously injected into the right axilla of nude mice to establish a tumor xenograft model. When tumor diameter reached 2–3 mm, mice were randomized into the following groups: control, ISO, NC inhibitor + ISO, and miR-874-3p inhibitor + ISO (*n* = 5 per group). Control animals received daily intraperitoneal injections of 0.5% DMSO, while the ISO group received 10 mg/kg ISO for 20 days. Concurrently, the miR-874-3p inhibitor (50 µL per injection) was administered via intratumoral multi-point injections twice weekly. Tumor length and width were measured on days 0, 5, 10, 15, 20, and 25 using calipers and an electronic scale. Tumor volume (V) was calculated as: V = 0.52 × length × width (mm^2^). Mice were euthanized, and tumor tissues were excised for subsequent experiments.

### Cell counting Kit-8 (CCK-8)

Cell viability and proliferation were measured using the CCK-8 assay. Briefly, cells were seeded in 96-well plates overnight and treated as indicated. Prior to detection, 10 µL of CCK-8 solution was added to each well, followed by 45 min incubation. Absorbance at 450 nm was measured using a microplate reader.

### Crystal violet staining

Cells were seeded in 96-well plates, allowed to adhere, then exposed to gradient ISO-containing medium for 48 h. Culture medium was discarded, cells washed with PBS, stained with 0.1% crystal violet for 15 min, rinsed with distilled water, decolorized with 10% acetic acid, and absorbance measured at 590 nm to reflect cell number changes.

### Colony formation

After treatment or transfection, cells were cultured in 6-well plates with fresh medium at 37 °C for 7 days. Colonies were fixed with methanol, stained with hematoxylin for 20 min, and manually counted using an optical microscope (Olympus, Japan).

### Intracellular and tissue ROS levels

Reactive oxygen species (ROS) were detected using the fluorescent probe 2′,7′-dichlorofluorescein diacetate (DCFH-DA; Sigma-Aldrich). DCFH-DA was dissolved in DMSO to prepare a stock solution. Cells were harvested by centrifugation and incubated with pre-warmed PBS containing DCFH-DA (10 µM) at 37 °C in the dark for 30 min. After washing twice with PBS, cells were resuspended in PBS. Flow cytometry (FITC [fluorescein isothiocyanate] channel) was performed in triplicate for each treatment. Data were analyzed using FlowJo software (FlowJo, USA; v10.0). For tumor tissues, sections were incubated with 10 µM DCFH-DA for 30 min, rinsed with PBS, and imaged using an FV10i laser scanning confocal microscope (Olympus, Japan).

### miRNA-Seq analysis

Total RNA (100 ng) isolated from breast cancer cells was subjected to miRNA expression profiling. RNA was labeled with Cy3 and amplified using the Low Input Quick Amp Labeling Kit (Agilent Technologies). Labeled RNA was hybridized to the Agilent 8 × 15 K Human miRNA Microarray Release 12.0 (catalog #G4872A). Small RNA sequencing was performed at BGI using the Illumina platform. Libraries were prepared using the TruSeq Small RNA Library Prep Kit (Illumina, USA). Sequencing was performed on an Illumina HiSeq 2000 (SE50 runs).

### qRT-PCR

Total RNA from the BF tissues and DT40 cells (control and IBDV-infected groups) was extracted using TRIzol reagent (Invitrogen) according to the manufacturer’s instructions. The purity and concentration of RNA samples were determined using Nanodrop 2000 (Thermo Fisher Scientific). Reverse transcription of mRNA and lncRNA was performed as previously described [39]; whereas that of miRNA was performed using miRcute miRNA First-strand cDNA Synthesis Kit (Tiangen, China). Further, RT-qPCR was performed as previously described [39]. Primer sequences are presented in Table 1. The relative mRNA levels of these genes were calculated using the 2 −ΔΔCt method, and β-actin or U6 was used as an internal control.

**Table 1:** qRT-PCR primer sequences

Gene	Forward (5′–3′)	Reverse (5′–3′)
hsa-miR-874-3p	CTGCCCTGGCCCGAGGGACCGA	General reverse primer
U6	GCCACGCAAATTCGTGAAGCGTTCCA	General reverse primer
PKM2	CGACTCTGAGGAGGAACAAGAA	CAGCAGAAGGTGATCCAGACT
HK2	TCCAGATGGGACAGAACAC	TGTCCGTTACTTTCACCCA
PGK1	TGGAACACGGAGGATAAAGTC	CAGGAAGGACTTTACCTTCCA
POU2F1	GACTCAAGAATGAACAATCCGT	CGAGTTGGACCTGATGGAG
β-actin	CACAGAGCCTCGCCTTTGCC	CACAGAGCCTCGCCTTTGCC

### Western blotting

Cells or tissues were lysed using RIPA lysis buffer, mixed appropriately, centrifuged, and the supernatant was collected and mixed with electrophoresis loading buffer. Proteins separated in the gel were transferred onto a PVDF membrane, followed by overnight incubation with primary antibodies. The next day, membranes were incubated with secondary antibodies and subsequently exposed and developed using a gel imaging system. The primary antibodies used included anti-POU2F1 (Abcam), anti-Caspase3/cleaved-caspase3 (Abclonal), anti-Bax (Abclonal), anti-BCL-2 (Abclonal), anti-PKM2 (Abclonal), anti-HK2 (Abclonal), anti-PGK1 (Abclonal), anti-Cleaved-Caspase 9 (Affinity Biosciences), anti-Cleaved-PARP (Affinity Biosciences) and anti-β-actin (Abclonal).

### Apoptosis analysis

The rate of apoptotic cells was analyzed using flow cytometry. Briefly, after treatment, cells from each group were stained with Annexin V-FITC and PI and analyzed using a Cytomics FC500 Flow Cytometry System (Beckman Coulter, Krefeld, Germany). Additionally, TUNEL staining (KeyGEN Biotech, China) was performed to assess apoptosis according to the manufacturer’s instructions.

### Mitochondrial membrane potential (JC-1)

Mitochondrial membrane potential (ΔΨm) was monitored using the Mitochondrial Membrane Potential Assay Kit with JC-1 (Beyotime). Breast cancer cells were incubated with JC-1 (10 µM final concentration) at 37 °C in the dark for 20 min. After washing with JC-1 buffer, red and green fluorescence of different groups were immediately observed under a fluorescence microscope.

### MitoSOX red probe assay

Cells were treated with ISO at gradient concentrations for the specified time, then incubated with 5 µM MitoSOX (Thermo Fisher) at 37 °C in the dark for 30 min. Subsequently, they were washed with PBS and immediately subjected to quantitative analysis by flow cytometry.

### Measurements of glucose consumption and lactate production

Glucose consumption and lactate production were measured in breast cancer cells using the Glucose Colorimetric Assay Kit (BioVision, Milpitas, USA) and Lactate Assay Kit (BioVision, Milpitas, USA), respectively. Glucose levels and lactate levels in the culture supernatants were quantified, and glucose consumption was calculated accordingly.

### ATP production assay

ATP content in cells was measured using an ATP Assay Kit (Abcam) according to the manufacturer’s protocol. Briefly, 100 µl of cell lysate was mixed with 100 µl of ATP reaction mixture and incubated for 30 min. Absorbance was measured at 570 nm using a microplate reader.

### SOD and CAT assays

Superoxide Dismutase (SOD) activity and Catalase (CAT) activity in cells were determined using the SOD Assay Kit and CAT Assay Kit (Nanjing Jiancheng), respectively, following the manufacturer’s instructions.

### Immunohistochemistry (IHC)

Tumor tissues were fixed with paraformaldehyde, embedded, and sectioned into 4 μm-thick slices. After deparaffinization and rehydration, antigen retrieval was performed using 10 mM sodium citrate solution. Sections were then incubated in 3% hydrogen peroxide solution for 15 min to block endogenous peroxidase activity. Subsequently, sections were incubated overnight at 4 °C with primary antibodies against Ki-67 or POU2F1 (Abcam), washed with PBS, and incubated with secondary antibodies for 30 min. Staining was visualized using 3,3’-diaminobenzidine (DAB).

### Luciferase reporter assay

A 3′-UTR fragment of POU2F1 (containing the predicted miR-874-3p binding site) was amplified from genomic DNA by PCR and cloned into the SacI and XhoI sites of the pmirGLO vector (Promega, USA) downstream of the luciferase gene sequence. A mutant construct containing the POU2F1 3′-UTR with deletion of the miR-874-3p seed region was synthesized by overlapping extension PCR and verified by sequencing.

For the luciferase activity assay, 2 × 10^4^ breast cancer cells were seeded in 24-well plates containing DMEM medium supplemented with 10% FBS 12 h prior to transfection. Cells were transfected with 100 ng of reporter plasmid (pmirGLO-POU2F1 wt 3′-UTR or pmirGLO-POU2F1 mut 3′-UTR), 20 nM miR-874-3p mimic or NC mimic, with or without ISO treatment. After 48 h, cells were harvested, and luciferase activity was analyzed using the Dual-Luciferase Reporter System (Promega) according to the manufacturer’s instructions. Luminescence signals were measured using a luminometer (GloMax 20/20, Promega), and firefly luciferase values were normalized to Renilla luciferase values.

Luciferase reporter plasmids containing the full-length promoter regions of PGK1, HK-2, and PKM2 were purchased from RiboBio (Guangzhou, China). These plasmids were co-transfected with a POU2F1 overexpression vector into breast cancer cells. Luciferase activity was analyzed using the Dual-Luciferase Reporter System (Promega) according to the manufacturer’s instructions.

### Statistical analysis

Data in this study were presented as mean±standard deviation. Data analysis was performed by Graphpad Prism (Version 7.0, USA) using a one-way analysis of variance (ANOVA). *P*-values of < 0.05 were considered significant.

## Results

### ISO inhibits breast cancer cell proliferation and induces apoptosis in vitro

**Fig. 1:**
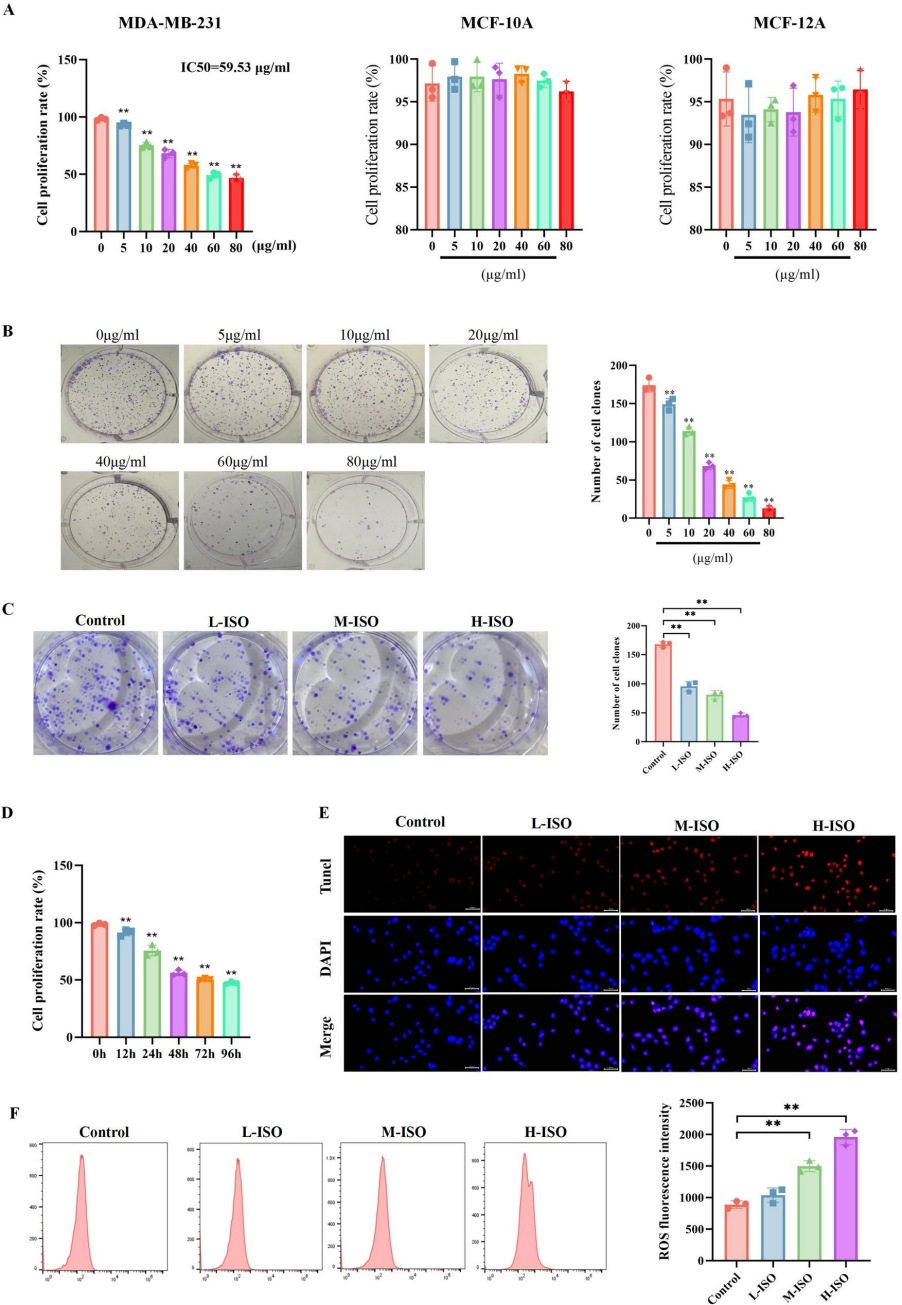
ISO inhibits breast cancer cell proliferation and promotes apoptosis. **A** MDA-MB-231, MCF-10 A and MCF-12 A cells were treated with different concentrations (0, 5, 10, 20, 40, 60, 80 µg/ml) of ISO for 48 h, followed by assessment of cell viability using the CCK8 assay. **B** The crystal violet staining method was used to determine the cell viability of MDA-MB-231 cells treated with different concentrations of ISO. **C** MDA-MB-231 cells were treated with low (20 µg/ml), medium (40 µg/ml), and high (60 µg/ml) concentrations of ISO for 48 h, and cell proliferation capacity was assessed using a colony formation assay. **D** MDA-MB-231 cells were treated with 60 µg/ml ISO for various durations (0, 12, 24, 48, 72, 96 h), and cell viability was measured using the CCK8 assay. **E** The effect of different concentrations of ISO on MDA-MB-231 cell apoptosis was detected using the TUNEL assay (bar = 100 μm). **F** Flow cytometry was used to detect the impact of different concentrations of ISO on ROS levels in MDA-MB-231 cells (DCFH-DA was used to label ROS). Mean ± SEM, *n* = 3, **p < 0.01. Statistical analysis was performed using one-way ANOVA.

To investigate the effect of ISO on renal cell carcinoma (RCC) cell growth, MDA-MB-231 cells were treated with different concentrations of ISO for 48 h, and cell proliferation was detected using the CCK8 method. ISO treatment significantly inhibited the proliferation of MDA-MB-231 cells in a dose-dependent manner (Fig. 1A). To validate the robustness of our findings and assess the specificity of ISO’s effects, we extended our investigation to normal breast cell lines. Specifically, we employed the same CCK8 method to detect cell proliferation in the MCF-10 A and MCF-12 A cell lines. The results indicated that ISO at any concentration did not affect the proliferation of these normal breast cells (Fig. 1A). We further corroborated the cell proliferation data obtained by CCK8 through the crystal violet staining method. This alternative staining approach yielded results entirely consistent with those from the CCK8 assay (Fig. 1B). To evaluate the anti-proliferative effects of ISO, we conducted colony formation assays and found that the proliferative capacity of cells in the ISO group decreased significantly compared to the control group, with reduced proliferation of MDA-MB-231 cells as the concentration of ISO increased (Fig. 1C). Further treatment with 60 µg/ml of ISO for varying durations revealed that the inhibitory effect of ISO on proliferation was also time-dependent (Fig. 1D). To study whether ISO induces apoptosis in breast cancer cells, MDA-MB-231 cells were exposed to different concentrations of ISO. TUNEL assay results showed that higher concentrations of ISO could induce more cell apoptosis (Fig. 1E). Additionally, flow cytometry results indicated that with increasing concentration, ISO induced higher levels of reactive oxygen species (ROS) (Fig. 1F). These results suggest that ISO can significantly inhibit cell growth in vitro, possibly related to its induction of higher ROS levels.

### ISO induces ROS-apoptosis while suppressing the Warburg effect

**Fig. 2:**
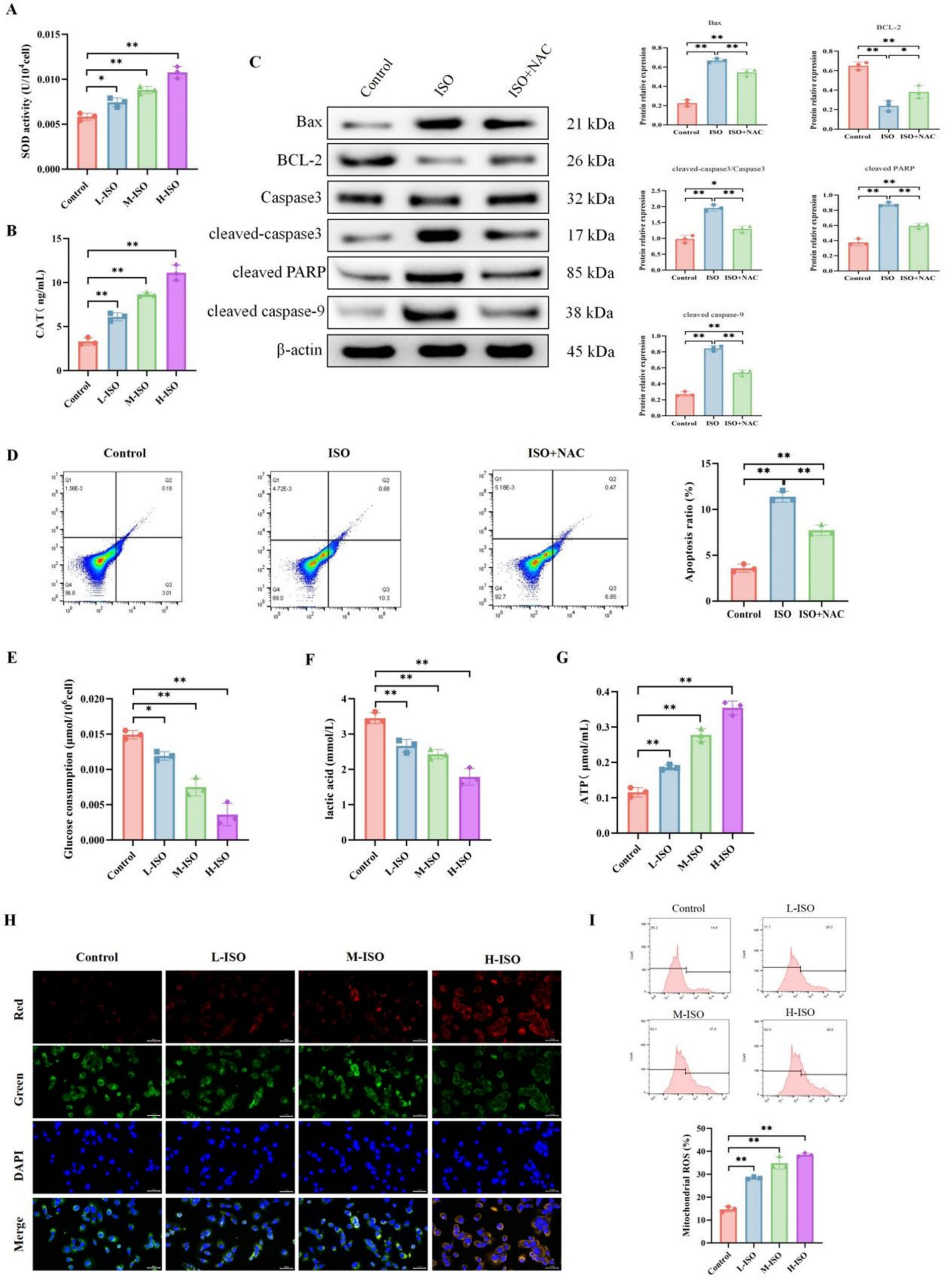
ISO induces ROS-apoptosis and suppresses the Warburg effect. MDA-MB-231 cells were treated with low (20 µg/ml), medium (40 µg/ml), and high (60 µg/ml) concentrations of ISO for 48 h, followed by detection of SOD and CAT activity using Superoxide Dismutase (SOD) assay kits (**A**) and Catalase (CAT) assay kits (**B**). Cells were pre-treated with 5 mM NAC for 1 h, then treated with 60 µg/ml ISO for 48 h, followed by Western blot detection of Bax, BCL-2, Caspase 3, cleaved-caspase 3, cleaved PARP and cleaved-caspase 9 (**C**), and detection of apoptosis rates using flow cytometry (**D**). MDA-MB-231 cells were treated with low (20 µg/ml), medium (40 µg/ml), and high (60 µg/ml) concentrations of ISO for 48 h, followed by detection of glucose consumption (**E**), lactate (**F**), and ATP (**G**) levels using commercial kits. JC-1 staining was performed to assess mitochondrial membrane potential. Higher membrane potential induces JC-1 aggregation into polymers, which emit red fluorescence (red channel), while lower membrane potential leads to the dispersion of JC-1 as monomers, which produce green fluorescence (green channel) (scale bar = 100 μm) (**H**). MDA-MB-231 cells were treated with 20/40/60 µg/ml ISO for 48 h, then stained with MitoSOX Red (to detect mitochondrial superoxide). Top: Flow cytometry histograms of MitoSOX fluorescence; Bottom: Quantified percentage of mitochondrial ROS (**I**). Mean ± SEM, *n* = 3, *p* < 0.01 (one-way ANOVA)

The “threshold” for triggering apoptosis via ROS in tumor cells is higher than in normal cells due to their robust antioxidant systems, which is a key factor contributing to treatment resistance in cancers. Therefore, we further analyzed the impact of ISO on critical antioxidant enzymes. As shown in Figs. 2A-B, the activities of SOD and CAT were found to be significantly increased in a concentration-dependent manner upon ISO treatment. This observation could reflect a compensatory response by tumor cells under oxidative stress. When intracellular ROS levels rise acutely, the primary antioxidant defense enzymes, SOD and CAT, may be subject to “stress-induced upregulation” in an attempt to restore redox homeostasis. Given the importance of ROS in apoptosis, the role of ROS in ISO-induced apoptosis was evaluated using the ROS scavenger N-acetyl-L-cysteine (NAC). Western blot analysis was used to detect changes in the expression of related proteins (Bax, BCL-2, Caspase 3, cleaved-caspase 3, cleaved PARP and cleaved-caspase 9). As shown in Fig. 2C, ISO treatment led to decreased expression of the anti-apoptotic protein Bcl-2 and increased expression of the pro-apoptotic protein Bax. Additionally, ISO treatment elevated the expression level of cleaved caspase-3, cleaved PARP and cleaved-caspase 9. Consistent with these results, Annexin V/PI staining and flow cytometry assays showed that ISO increased the percentage of apoptotic cells (Fig. 2D). However, the changes in expression levels of apoptosis-related proteins and the number of apoptotic cells detected by flow cytometry caused by ISO were reversed by NAC.

Metabolic reprogramming such as the Warburg effect in tumor cells profoundly affects ROS production and NADPH supply; however, the role of ISO in regulating cancer cell metabolism remains unclear. Thus, we investigated whether ISO-induced ROS and apoptosis are associated with cellular metabolic reprogramming. We found that ISO treatment suppressed lactate levels and glucose consumption in breast cancer cells (Fig. 2E–F). Further JC-1 staining analysis revealed enhanced mitochondrial membrane potential in ISO-treated cells (Fig. 2H), indicating significantly increased mitochondrial function in breast cancer cells post-ISO application. This finding was further supported by detection of increased ATP production in ISO-treated breast cancer cells, indicating enhanced mitochondrial oxidative phosphorylation activity (Fig. 2G). Meanwhile, MitoSOX probe detection showed that mitochondrial ROS levels in breast cancer cells were elevated in an ISO concentration-dependent manner, with the highest level observed under high-dose ISO treatment (Fig. 2I). As a key intracellular signaling molecule, ROS has been shown to modulate microRNA (miRNA) expression by either regulating epigenetic modification enzyme activity or directly affecting transcription factor function. This suggests that ISO-induced ROS accumulation may similarly reshape the miRNA expression profile through analogous mechanisms. In summary, these findings suggest that ISO treatment suppresses the Warburg effect in cancer cells, leading to restored mitochondrial function and increased ATP and ROS production, potentially promoting apoptosis and inhibiting cancer cell proliferation.

### miR-874-3p mediates the anti-breast cancer effects of ISO

**Fig. 3:**
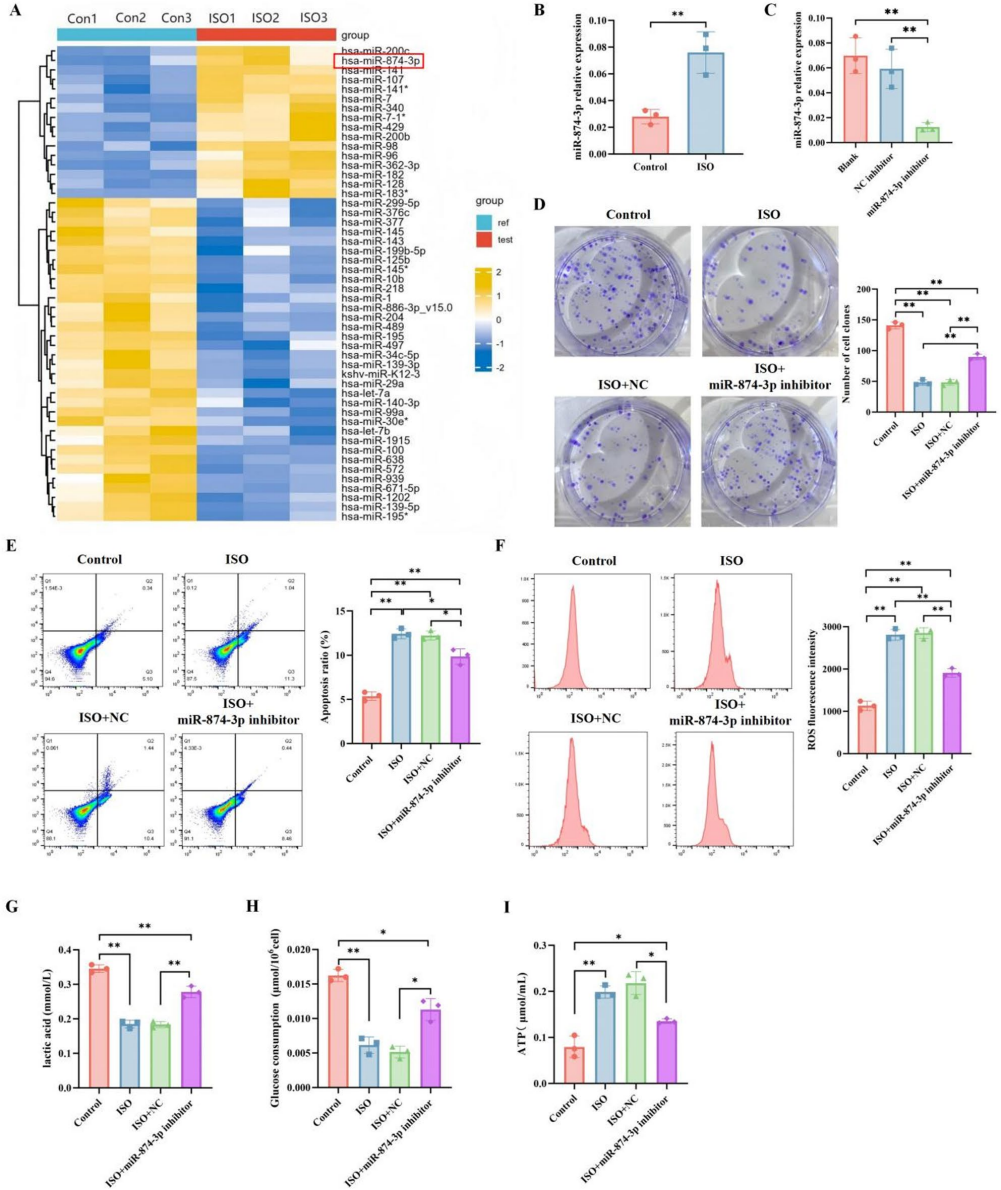
miR-874-3p mediates the anti-breast cancer effects of ISO. **A** Differential expression of miRNAs between Control and ISO-treated MDA-MB-231 cells with three biological replicates per group analyzed by miRNA-seq. **B** Expression change of miR-874-3p in MDA-MB-231 cells detected by qRT-PCR after treatment with 60 µg/ml ISO for 48 h. **C** Transfection efficiency detected by qRT-PCR after transfecting MDA-MB-231 cells with miR-874-3p inhibitor or NC inhibitor. After cell transfection, cells were treated with 60 µg/ml ISO for 48 h, then cell proliferation ability was detected by colony formation assay (**D**), apoptosis rate by flow cytometry (**E**), ROS levels by flow cytometry (**F**), and glucose consumption (**G**), lactate (**H**), and ATP (**I**) levels using commercial kits. Mean ± SEM, *n* = 3, *p < 0.05, **p < 0.01. Statistical analysis was conducted by on ANOVA

To explore the potential molecular mechanisms underlying ISO’s inhibition of breast cancer, we obtained differential miRNAs potentially affected by ISO through miRNA transcriptome sequencing analysis (Fig. 3A). Among them, miR-874-3p was significantly upregulated in the ISO group. miR-874-3p has been shown to have an inhibitory effect on various cancers, including breast cancer. qRT-PCR results were consistent with the transcriptome sequencing results, showing that ISO treatment significantly upregulated miR-874-3p expression (Fig. 3B). Therefore, based on its differential expression identified by high-throughput screening and well-documented functional relevance in breast cancer, miR-874-3p was selected as the target for subsequent mechanistic investigations.

To investigate whether miR-874-3p mediates the anti-tumor effects induced by ISO, MDA-MB-231 cells were transfected with either a normal control (NC) or hsa-miR-874-3p inhibitor and subsequently treated with ISO for further experiments, compared with the NC inhibitor group, the expression level of miR-874-3p was significantly reduced in the miR-874-3p inhibitor group, indicating that transfection successfully suppressed miR-874-3p expression (Fig. 3C). According to the results, colony formation assays confirmed that the miR-874-3p inhibitor abolished ISO’s ability to inhibit the proliferation of breast cancer cells (Fig. 3D). In addition to proliferation, miR-874-3p also mediates ISO-induced changes in reactive oxygen species (ROS), apoptosis, and glycolysis. Flow cytometry results showed that when the miR-874-3p inhibitor was present, ISO-induced apoptosis and ROS levels significantly decreased (Fig. 3E–F). Moreover, the miR-874-3p inhibitor also reversed ISO’s regulatory effects on ATP, glucose consumption, and lactate levels (Fig. 3G–I). In summary, ISO’s effects on proliferation, apoptosis, ROS, and glycolysis in breast cancer cells are mediated by miR-874-3p.

### POU2F1 as a target gene of miR-874-3p in ROS apoptosis and warburg effect in breast cancer

**Fig. 4:**
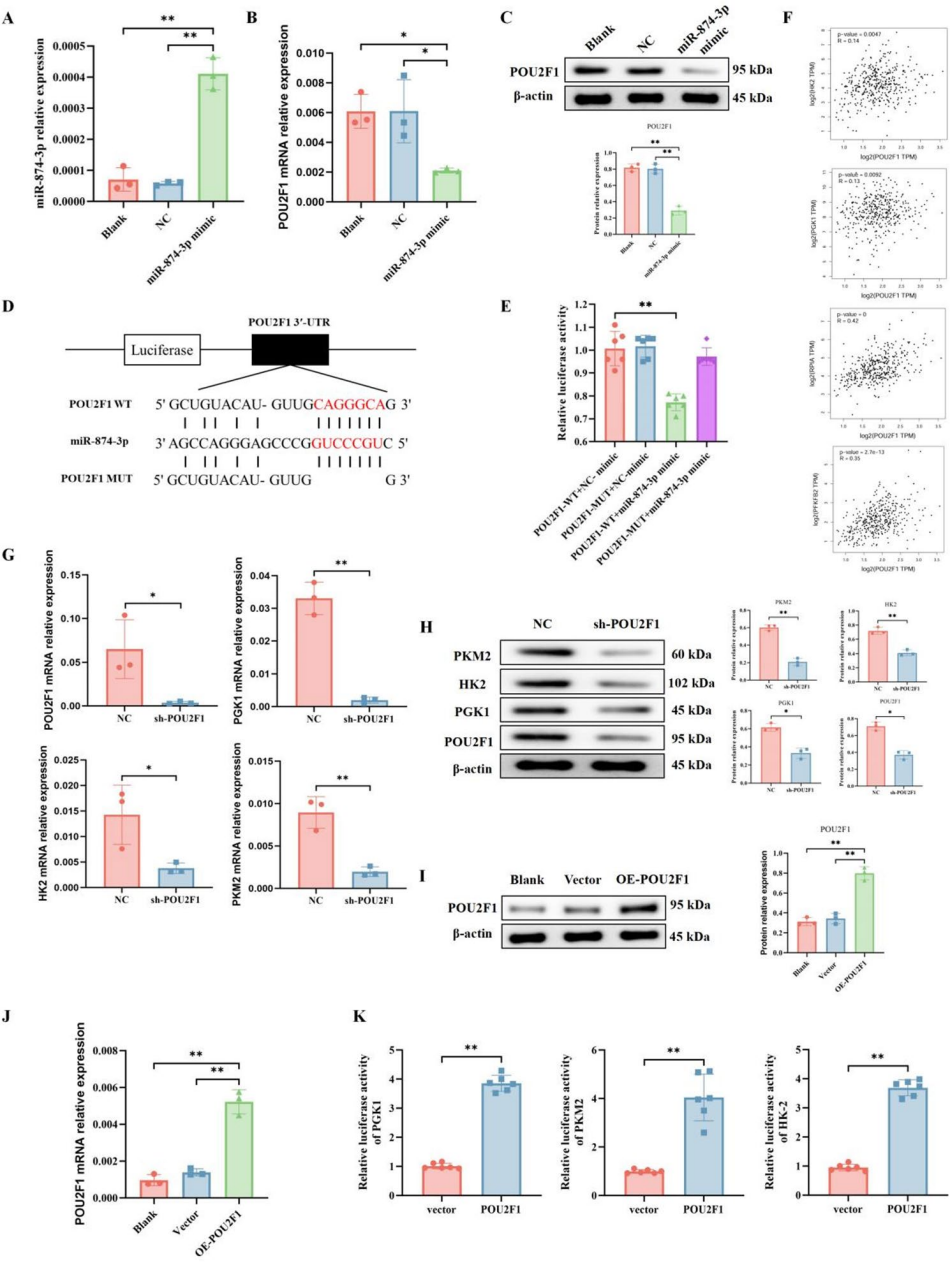
POU2F1 is a target gene of miR-874-3p. MDA-MB-231 cells were transfected with miR-874-3p mimic or NC mimic, and the levels of miR-874-3p and POU2F1 mRNA were measured by qRT-PCR (**A**–**B**). The protein level of POU2F1 was assessed by Western blot (**C**). A binding site for miR-874-3p was identified in the 3’-UTR region of POU2F1 (**D**). The targeting relationship between miR-874-3p and POU2F1 was analyzed using a dual-luciferase reporter assay (**E**). Analysis via the GEPIA database revealed that POU2F1 expression positively correlates with the levels of hexokinase 2 (HK2), phosphoglycerate kinase 1 (PGK1), 6-phosphofructo-2-kinase/fructose-2,6-biphosphatase 2 (PFKFB2), and ribose-5-phosphate isomerase A (RPIA) in breast cancer tissues. TPM: Transcripts Per Million. (**F**) MDA-MB-231 cells were transfected with sh-POU2F1 or NC, and the expression levels of PGK1, HK2, and PKM2 were detected by qRT-PCR (**G**) and Western blot (**H**). Overexpression of POU2F1 (OE-POU2F1) or Vector in MDA-MB-231 cells was followed by measurement of POU2F1 levels by qRT-PCR (**J**) and Western blot (**I**). **K** MDA-MB-231 cells were co-transfected with luciferase reporter plasmids containing the promoters of PGK1, HK2, or PKM2 along with either Vector or POU2F1 overexpression constructs. Luciferase activity was measured to evaluate the effect of POU2F1 on the promoter activities of PGK1, HK2, and PKM2. Mean ± SEM, *n* = 3, *p < 0.05, **p < 0.01. Statistical analysis was performed using one-way ANOVA.

POU domain class 2 transcription factor 1 (POU2F1), also known as octamer transcription factor 1 (OCT1), is considered a potential prognostic marker for various epithelial cancers, including breast cancer. Analysis of POU2F1 protein levels in primary breast cancer tissues reveals higher expression compared to normal tissues [20]. Functionally, the inhibition of POU2F1 reduces the metastatic potential, hypoxia resistance, and drug resistance of metastatic breast cancer cells [21]. Importantly, Starbase analysis (http://starbase.sysu.edu.cn/index.php) indicates that POU2F1 has binding sites for miR-874-3p. Therefore, we hypothesize an association between this interaction and breast cancer. Successful transfection of miR-874-3p mimic into cells reduced POU2F1 mRNA and protein expression levels in vitro (Fig. 4A–C). Subsequent Starbase predictions revealed that miR-874-3p can target the 3’-UTR region of POU2F1 (Fig. 4D). Moreover, transfection with miR-874-3p mimic, but not mimic NC, significantly decreased cell luciferase intensity driven by WT-POU2F1 (Fig. 4E). Thus, miR-874-3p inhibits POU2F1 expression in breast cancer cells through direct targeting.

Research has shown that POU2F1 is closely related to cancer cell metabolic reprogramming, and this function depends on its transcriptional regulatory activity [22]. To understand the mechanism by which POU2F1 enhances glycolysis, we analyzed the potential correlation between POU2F1 and glycolysis-related genes on the GEPIA website. We found that POU2F1 transcripts are positively correlated with hexokinase 2 (HK2), phosphoglycerate kinase 1 (PGK1), 6-phosphofructo-2-kinase/fructose-2,6-bisphosphatase 2 (PFKFB2), and ribose-5-phosphate isomerase A (RPIA) levels in breast cancer tissues (Fig. 4F). Interestingly, after transfection of sh-POU2F1 or NC into MDA-MB-231 cells, qRT-PCR and Western blot analyses showed that the expression levels of glycolysis-related genes PGK1, HK-2, and pyruvate kinase M1/2 (PKM2) were significantly reduced (Fig. 4G–H). Further analysis using the Jaspar website indicated that POU2F1 could potentially bind to the promoters of PGK1, HK-2, and PKM2. Transfection of the POU2F1 expression plasmid (Fig. 4I–J) and luciferase reporter plasmids containing human PGK1, HK-2, or PKM2 promoter sequences revealed that POU2F1 overexpression significantly increased the luciferase activity regulated by the PGK1, HK-2, or PKM2 promoters in breast cancer cells (Fig. 4K). These results indicate that POU2F1 promotes the transcription of PGK1, HK-2, and PKM2.

**Fig. 5:**
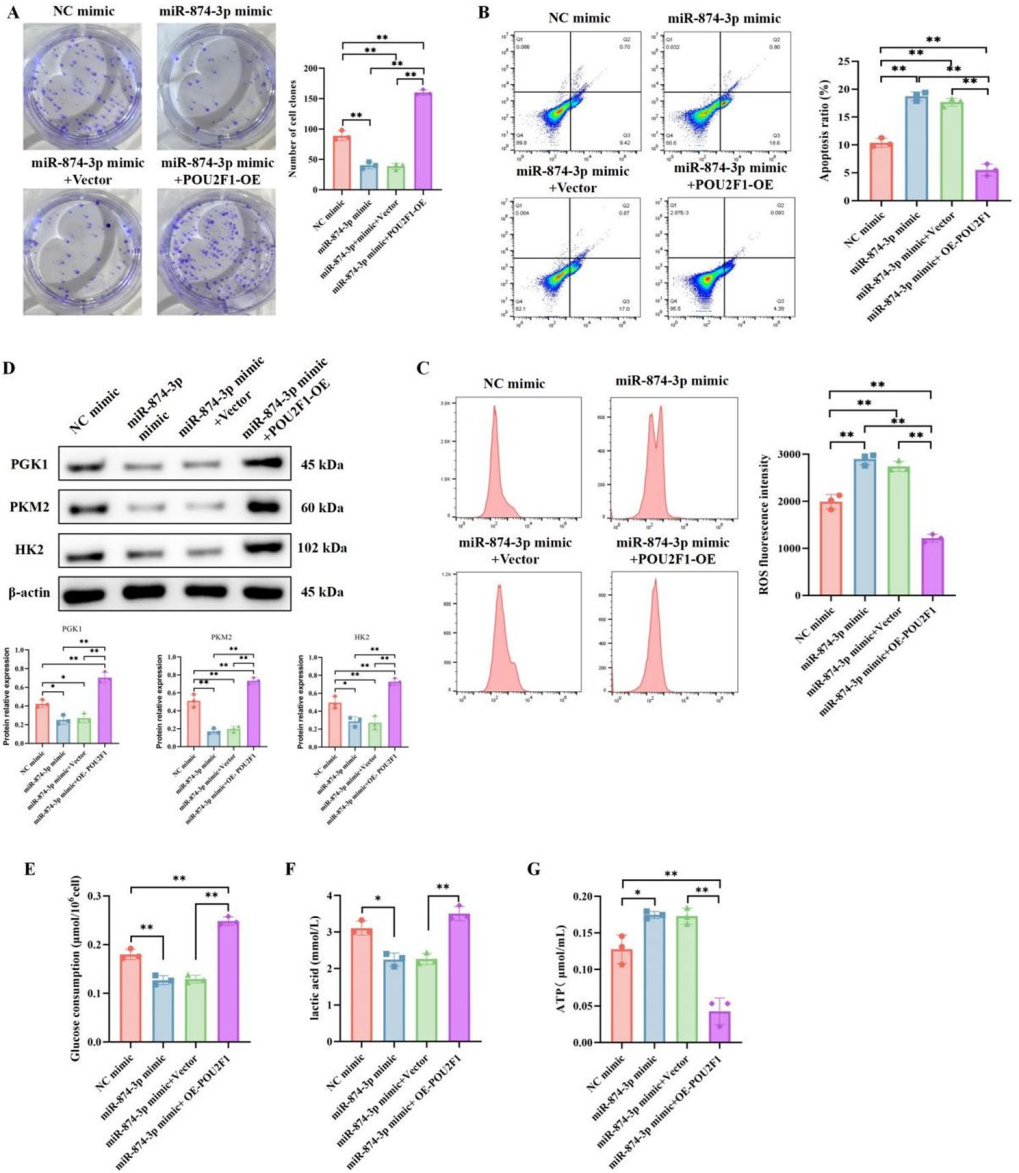
miR-874-3p regulates ROS-mediated apoptosis and the Warburg effect in breast cancer through POU2F1. MDA-MB-231 cells were transfected with miR-874-3p mimic alone or co-transfected with Vector and OE-POU2F1. Cell proliferation was assessed by colony formation assay (**A**), while apoptosis and ROS levels were evaluated by flow cytometry (**B**–**C**). The expression levels of PGK1, HK2, and PKM2 were measured by Western blot (**D**). Glucose consumption, lactate production, and ATP levels were determined using commercial assay kits (**E**–**G**). Mean ± SEM, *n* = 3, *p < 0.05, **p < 0.01. Statistical analysis was performed using one-way ANOVA

To demonstrate whether this regulatory mechanism mediates the effect of miR-874-3p on apoptosis and glycolysis in breast cancer, we conducted rescue experiments by transfecting POU2F1 overexpression plasmids into cells transfected with miR-874-3p mimic. The results showed that miR-874-3p mimic inhibited cell proliferation, while promoting ROS levels and apoptosis; however, POU2F1 overexpression significantly reversed the regulatory effects of miR-874-3p mimic on proliferation, ROS, and apoptosis (Fig. 5A–C). Additionally, POU2F1 overexpression eliminated the impact of miR-874-3p mimic on cellular ATP, glucose consumption, and lactate levels (Fig. 5D–G). This suggests that POU2F1 mediates the regulation of ROS, apoptosis, and glycolysis by miR-874-3p in breast cancer.

### Restoration of POU2F1 expression suppresses the anti-breast cancer effects of ISO

**Fig. 6:**
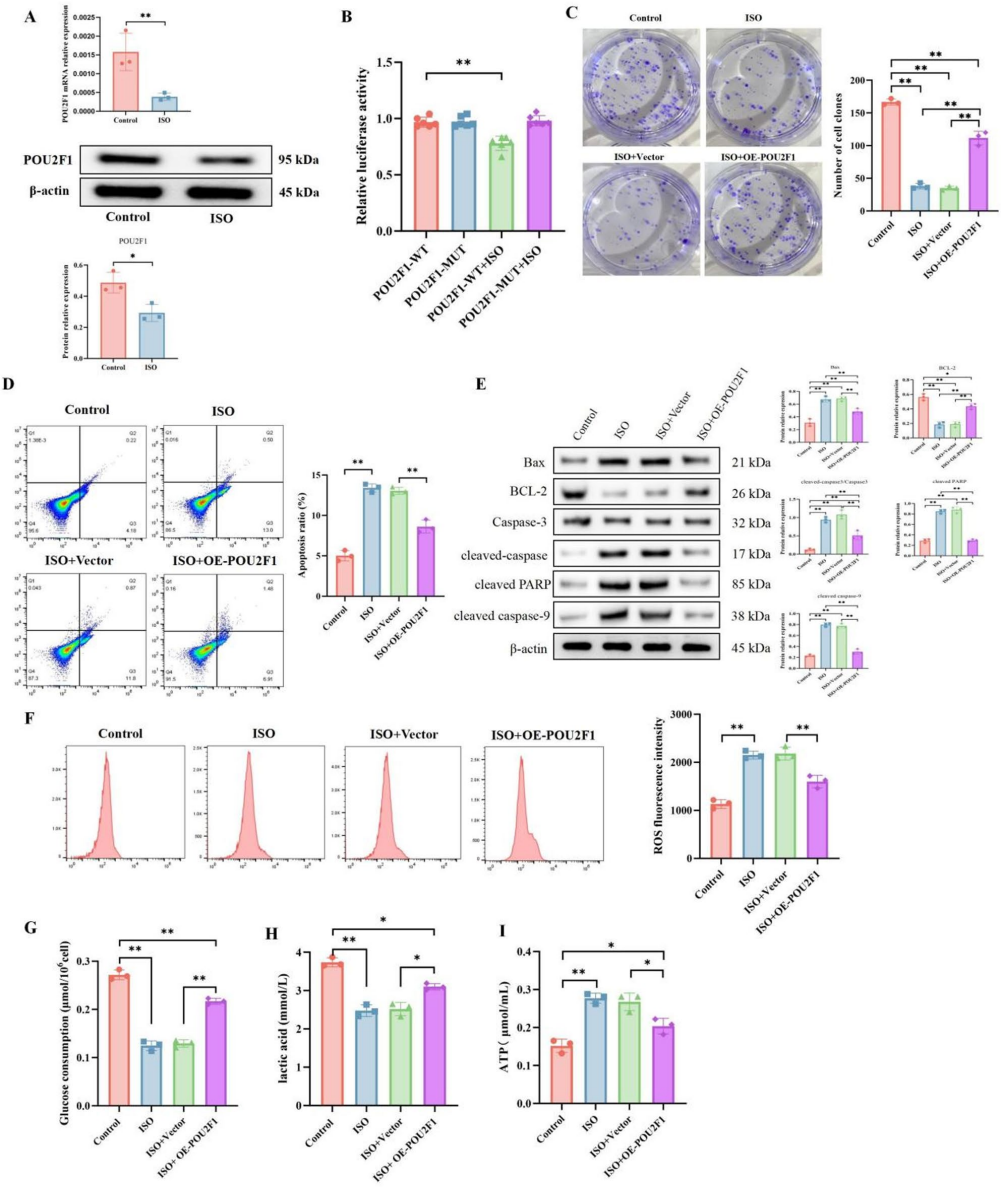
Restoration of POU2F1 expression suppresses the anti-breast cancer effects of ISO. Cells were treated with 60 µg/ml ISO for 48 h, and POU2F1 expression was detected using qRT-PCR and Western blot (**A**). The effect of ISO on POU2F1 luciferase reporter vector activity was assessed using dual-luciferase assays (**B**). In MDA-MB-231 cells, OE-POU2F1 or Vector was transfected, followed by treatment with 60 µg/ml ISO for 48 h. Cell proliferation was evaluated using colony formation assays (**C**); apoptosis and ROS levels were measured using flow cytometry (**D**, **F**); Caspase-3, cleaved-Caspase-3, Bax, and Bcl-2 protein expressions were determined using Western blot (**E**); glucose consumption, lactate production, and ATP levels were assessed using commercial kits (**G**–**I**). Mean ± SEM, *n* = 3, *p < 0.05, **p < 0.01. Statistical analysis was performed using one-way ANOVA

Given that the ROS levels and Warburg effect influenced by ISO are associated with breast cancer progression, and we have previously demonstrated that miR-874-3p can directly regulate POU2F1 in breast cancer cells, we hypothesized that miR-874-3p-mediated regulation of POU2F1 expression is involved in the inhibitory effects of ISO on breast cancer. To test this hypothesis, we first examined the expression of POU2F1 in breast cancer cells with or without ISO treatment. As shown, compared to untreated controls, ISO treatment led to a reduction in both mRNA and protein levels of POU2F1 in cultured cells (Fig. 6A). Additionally, ISO suppressed the luciferase activity of the wild-type POU2F1 3 ′-UTR but did not affect the luciferase activity of the mutant POU2F1 3 ′-UTR (Fig. 6B). These results further indicate that POU2F1 is a direct target of miR-874-3p in breast cancer cells and that ISO treatment enhances miR-874-3p-mediated suppression of POU2F1 expression.

Next, a similar strategy was used to explore the relationship between POU2F1 and ISO-mediated tumor cell inhibition. Cells transfected with OE-POU2F1 were treated with ISO. OE-POU2F1 transfection abolished ISO’s inhibitory effect on cell proliferation capacity (Fig. 6C), an effect similar to that observed with miR-874-3p silencing. Moreover, OE-POU2F1 transfection restored changes in apoptosis (Fig. 6D–E), ROS increase (Fig. 6F), and glycolysis alterations (Fig. 6G–I) induced by ISO treatment. These results suggest that POU2F1 may also act as a downstream effector of ISO in breast cancer.

### ISO regulating breast cancer progression via the miR-874-3p/POU2F1 axis in vivo

**Fig. 7:**
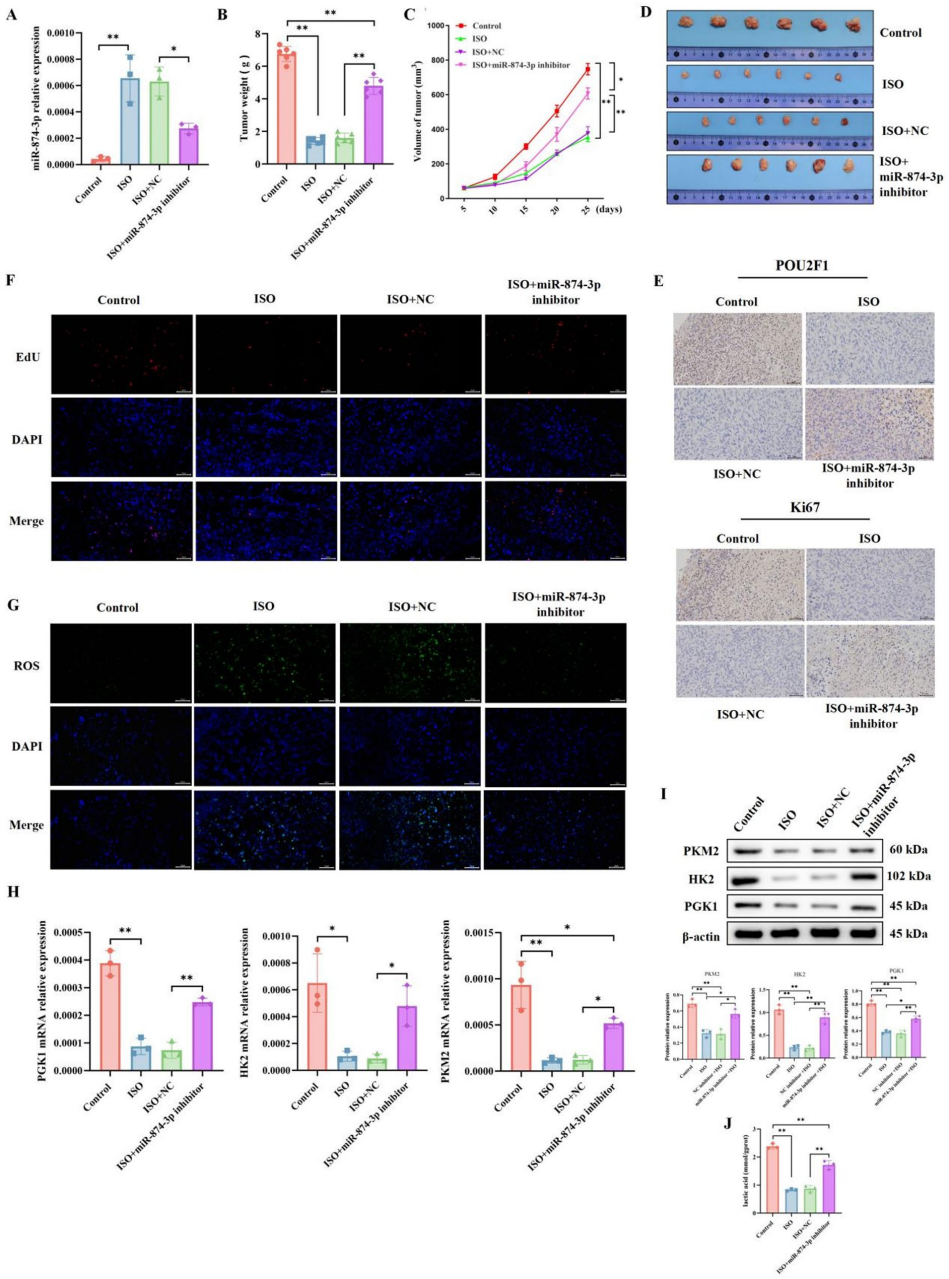
ISO regulates breast cancer progression via the miR-874-3p/POU2F1 axis. Levels of miR-874-3p in tumor tissues were detected by qRT-PCR (**A**); Quantitative analysis of tumor weight (**B**); Average growth curves of subcutaneous xenograft tumors in each group of nude mice (**C**); Representative images of tumors from each group of mice (**D**); Immunohistochemical staining for Ki-67 and POU2F1 in tumors from each group of mice, bar = 50 μm (**E**); Fluorescence microscopy observation of EdU-positive cells after tail vein injection of EdU before euthanasia, bar = 50 μm (**F**); Fluorescence microscopy observation of ROS levels after DCFH-DA treatment of tumor tissue sections, bar = 50 μm (**G**); Detection of PGK1, HK2,and PKM2 expression in tumor tissues by qRT-PCR and Western blot (**I**–**H**); Detection of lactate levels in tumor tissues using a commercial kit (**J**). Mean ± SEM, *n* = 6, *p < 0.05, **p < 0.01. Statistical analysis was conducted by one-way ANOVA

To investigate the roles of ISO and the miR-874-3p/POU2F1 axis in tumor growth in vivo, we further validated the results obtained from in vitro experiments. Xenograft mice were treated with ISO while miR-874-3p levels were manipulated through intratumoral injection of a miR-874-3p inhibitor (Fig. 7A). Evaluation of the growth, size, and volume of subcutaneous xenograft tumors revealed that ISO treatment inhibited tumor growth; however, the application of the miR-874-3p inhibitor attenuated the tumor-suppressive effects of ISO (Fig. 7B–D).

Given that POU2F1 is a downstream target of both ISO and miR-874-3p, we assessed POU2F1 expression in tumor tissues using immunohistochemistry (IHC). The results showed that ISO intervention reduced POU2F1 expression in tumor tissues, whereas the miR-874-3p inhibitor promoted its expression. Furthermore, cell proliferation capacity was evaluated by IHC (using Ki67 antibody) and EdU assays. The application of ISO decreased the number of Ki67-positive or EdU-positive cells, but the miR-874-3p inhibitor enhanced cell proliferation (Fig. 7E–F).

Additionally, ROS levels in tumor tissues were examined using a ROS probe. The results indicated that ISO increased ROS levels in tumor tissues, whereas the miR-874-3p inhibitor almost completely abolished the regulatory effect of ISO on ROS (Fig. 7G). To further validate the in vitro findings, we also measured the Warburg effect in tumor tissues. ISO significantly suppressed the levels of Warburg effect-related factors PGK1, HK2, PKM2 mRNA, protein, and lactate in tumor tissues, while the miR-874-3p inhibitor upregulated these indicators (Fig. 7I–J). Thus, ISO can inhibit tumor growth and the Warburg effect in vivo via the miR-874-3p/POU2F1 axis.

## Discussion

Despite a recent decline in the mortality rate of breast cancer in affluent countries, the incidence and mortality rates in less developed regions continue to be alarmingly high, primarily due to inadequate early detection and limited access to appropriate treatment facilities [23]. Consequently, the development of more effective therapeutic strategies is imperative. Isoimperatorin (ISO) has shown significant potential in the treatment of various malignancies, including breast cancer. For instance, molecular docking studies have shown that ISO exhibits inhibitory effects on multiple targets, such as estrogen receptor α (ERα), progesterone receptor (PR), epidermal growth factor receptor (EGFR), and mammalian target of rapamycin (mTOR) [19]. In vitro experiments further validated its potential anti-breast cancer activity, suggesting that ISO may exert its anticancer effects through a multi-target mechanism. Here, we observed that ISO could inhibit breast cancer cell proliferation and the Warburg effect while promoting reactive oxygen species (ROS) production and apoptosis both in vivo and in vitro. Additionally, ISO treatment led to increased expression of miR-874-3p and reduced mRNA and protein levels of POU2F1 in breast cancer cells and tumor tissues derived from these cells. Functionally, ISO treatment enhanced miR-874-3p-mediated suppression of POU2F1 expression.

Aberrant levels of microRNAs (miRNAs) in malignant tumors have garnered increasing attention, and changes in their expression have been shown to suppress tumorigenesis both in vitro and in vivo. However, the molecular mechanisms underlying miRNA expression modulation require further exploration. Recent studies have reported the role of ISO in miRNA regulation. For example, ISO reduces melanin content via the miR-3619/CSTB and miR-3619/CSTD axes [24]. Dysregulation of miR-874-3p has been implicated in various diseases, including intestinal barrier dysfunction, skeletal metabolism disorders, allergic rhinitis, asthma, and cognitive impairments [25, 26], as well as the progression and metastasis of multiple malignancies [27, 28]. Notably, miR-874-3p is downregulated in breast cancer, and its overexpression inhibits malignant behaviors of breast cancer cells [29]. Furthermore, miR-874-3p suppresses breast cancer cell proliferation, colony formation, and metastasis by targeting cyclin E1 (CCNE1) [30]. Our study is the first to demonstrate that ISO treatment upregulates miR-874-3p expression. Functionally speaking, silencing miR-874-3p abrogated the effects of ISO on breast cancer cell proliferation, ROS levels, apoptosis, and the Warburg effect. Thus, we conclude that ISO exerts its antitumor effects in vitro by upregulating miR-874-3p. Bioinformatics prediction was employed to identify potential downstream target genes of miR-874-3p, with POU2F1 ultimately selected for further investigation. POU2F1 belongs to the POU domain family and plays crucial roles in various biological processes, including cell proliferation, differentiation, and survival [31]. Recent studies have shown that POU2F1 is expressed in certain solid tumors, including breast cancer, and can predict patient prognosis [22, 32, 33]. For instance, POU2F1 is upregulated in gastric cancer and promotes tumor metastasis by directly targeting PIK3R1, PIK3R3, and the PI3K/Akt/mTOR signaling pathway via miR-29b-3p/miR-29a-3p [34]. Previous research has demonstrated that knockdown of POU2F1 inhibits breast cancer cell migration, reduces the expression of invasion- and metastasis-related genes, and increases cell death associated with hypoxia or chemotherapeutic agents [21]. In this study, ISO treatment not only upregulated miR-874-3p expression but also suppressed POU2F1 levels.

Previous studies have demonstrated that POU2F1 is closely associated with the reprogramming of glucose metabolism in tumors. Whether by directly modulating the activity of glycolysis-related enzymes or disrupting energy metabolic pathways through other means, inhibition of aerobic glycolysis in tumor cells effectively enhances the accumulation of intracellular reactive oxygen species (ROS), thereby inducing apoptosis. This strategy offers a potential direction for developing novel anticancer therapies. For instance, when glycolysis is inhibited using drugs such as 2-deoxy-D-glucose (2DG), the intracellular NADPH levels in tumor cells are significantly reduced, disrupting glutathione (GSH) homeostasis and rendering cells more susceptible to other treatment modalities, such as radiotherapy or chemotherapy [35]. Melatonin-induced suppression of glycolysis in renal cancer cells has been associated with elevated ROS levels and ROS-dependent apoptosis [36]. Hemistepsin A (HsA), a PDK1 inhibitor, increases mitochondrial ROS content in colorectal cancer cells by reducing lactate production and enhancing oxygen consumption rates, thereby triggering ROS-dependent apoptotic pathways [37]. In this study, ISO induced an increase in ROS levels and apoptosis in breast cancer cells, potentially due to its regulation of miR-874-3p and POU2F1, which subsequently inhibits the Warburg effect.

POU2F1, a transcription factor, is closely associated with poor prognosis and chemoresistance when overexpressed in various cancers [22]. Research has shown that POU2F1 promotes tumor cell proliferation, invasion, and metabolic reprogramming by directly regulating the expression of glycolysis-related genes [38]. Mechanistically, POU2F1 binds to the promoter regions of target genes, enhancing their transcriptional activity and driving aerobic glycolysis, which is crucial for tumor cell survival. In line with previous studies, our findings confirm that POU2F1 promotes the transcription of glycolysis-related genes, such as PGK1, HK-2, and PKM2, in breast cancer cells. Consequently, inhibiting the expression or function of POU2F1 can effectively disrupt glycolysis-related metabolic pathways, thereby suppressing tumor cell proliferation and invasiveness. Significantly, dual-luciferase reporter assays have verified the interaction between miR-874-3p and POU2F1. Moreover, the overexpression of POU2F1 reversed the inhibitory effects of ISO on tumor progression, suggesting that the miR-874-3p/POU2F1 axis plays a key role in mediating the anti-breast cancer efficacy of ISO. This study is the first to comprehensively elucidate the mechanism of action of the miR-874-3p/POU2F1 axis through both in vitro and in vivo experiments. In subsequent research, we will collect more data to validate and refine these findings.

The miR-874-3p/POU2F1 axis mediated by ISO has been identified as a potential pathway regulating cell proliferation and metabolic reprogramming both in vitro and in vivo, underscoring its critical role in the pathogenesis of breast cancer. However, further experiments are necessary to comprehensively elucidate the mechanisms by which ISO regulates miR-874-3p expression, which will clarify the pharmacological mechanisms of ISO. In summary, we provide evidence that ISO inhibits POU2F1 expression by increasing miR-874-3p levels, thereby suppressing breast cancer progression. These findings not only present new therapeutic options but also enhance our understanding of the functional mechanisms of ISO.

## Supplementary Information

Below is the link to the electronic supplementary material.

Supplementary Material 1

## Data Availability

All data utilized in this work could be obtained from the corresponding author upon request.
