# Supplementary material for: Isoimperatorin suppresses triple-negative breast cancer by modulating miR-874-3p/POU2F1 axis: a new avenue for metabolic and redox intervention
Source: Biol Res. 2026 Mar 12;59:23. doi: 10.1186/s40659-026-00678-x (PMC13097874; doi:10.1186/s40659-026-00678-x)

**Figure 2 C**

Bax


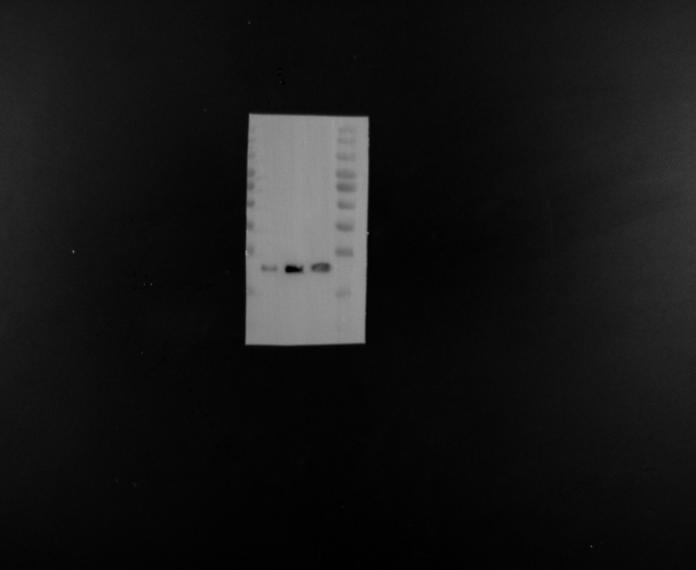


BCL-2


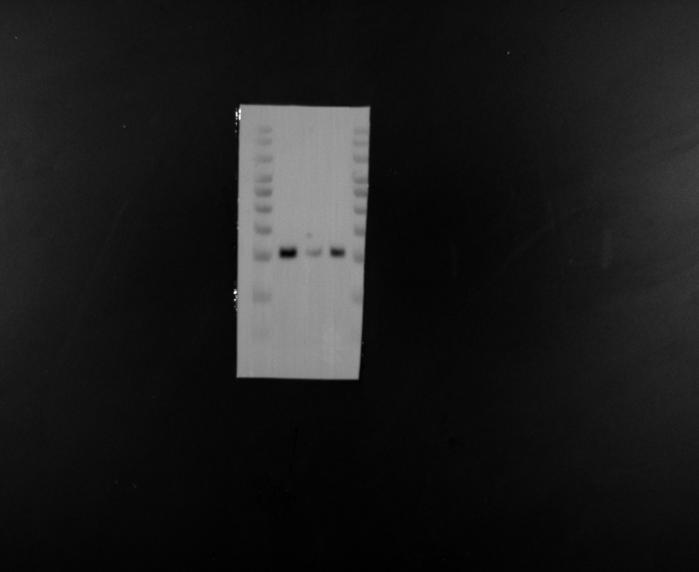


Caspase-3


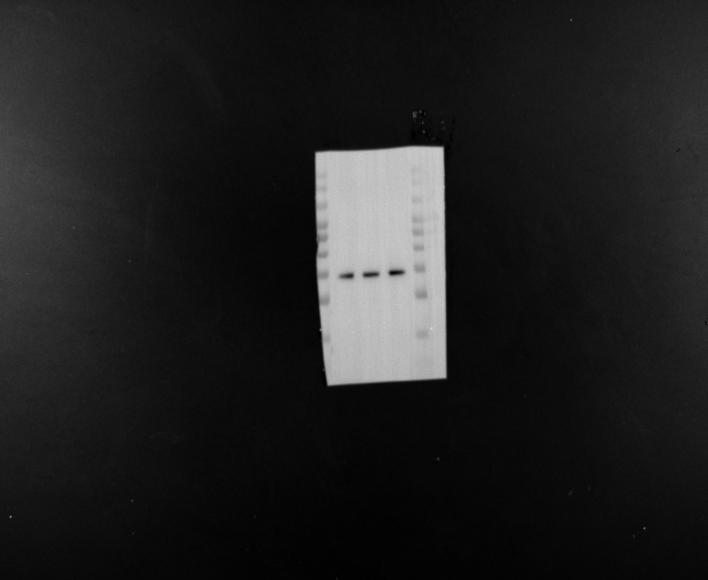


Cleaved-casepase 3


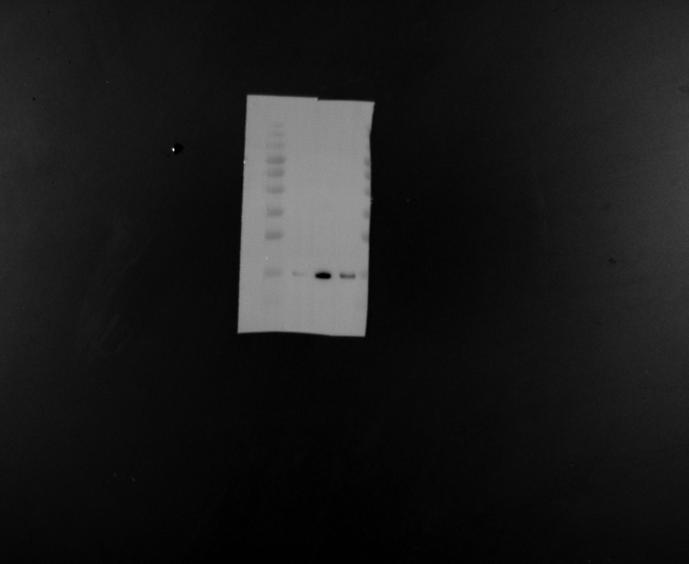


β-actin


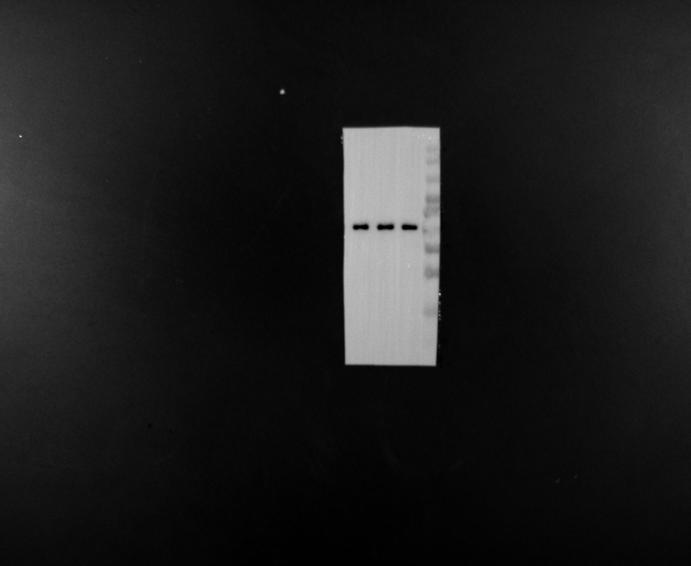


**Figure4 C**

POU2F1

**
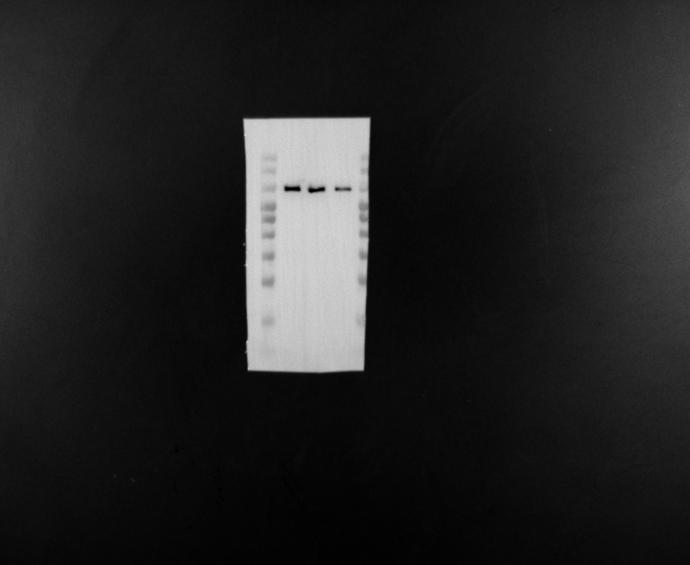
**

β-actin

**
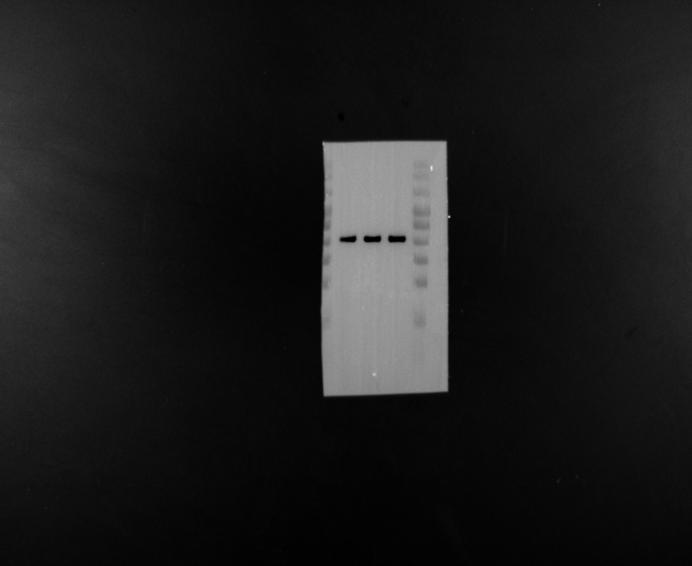
**

**Figure4 H**

PGK1

**
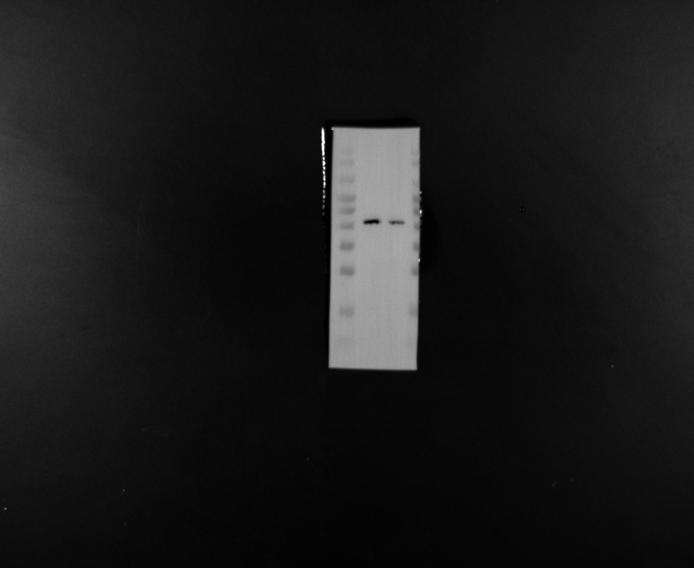
**

HK2

**
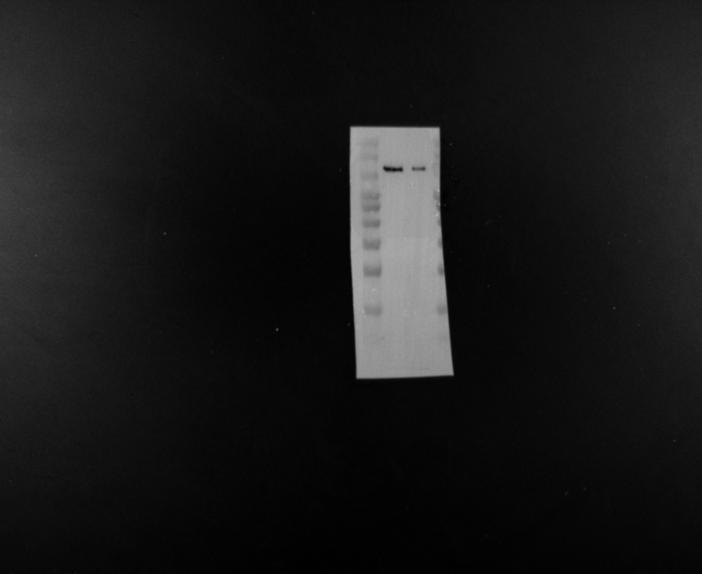
**

PKM2

**
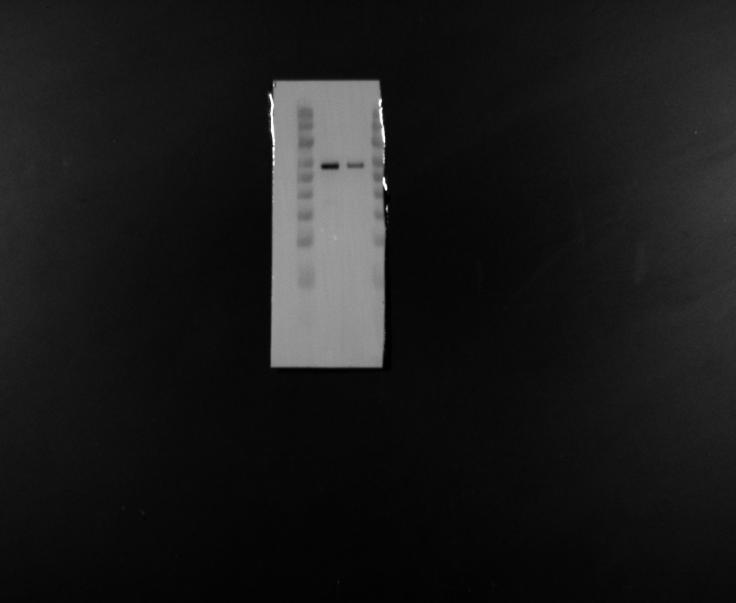
**

POU2F1

**
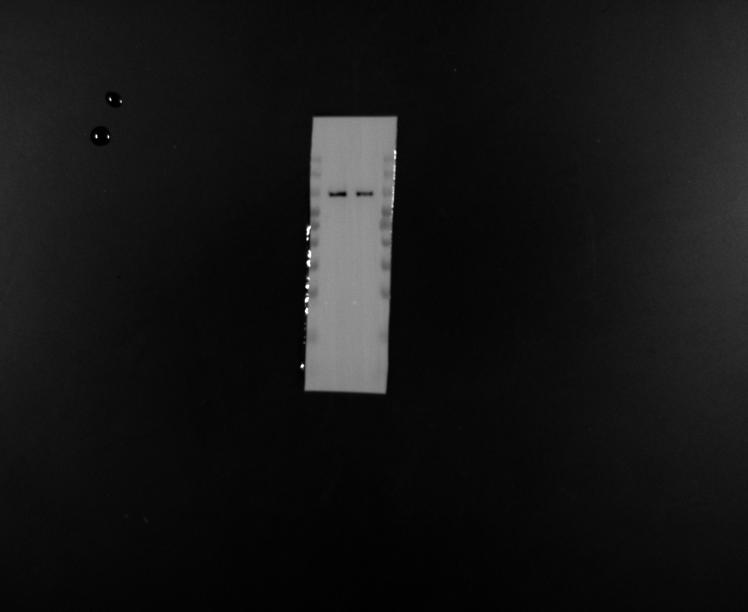
**

β-actin

**
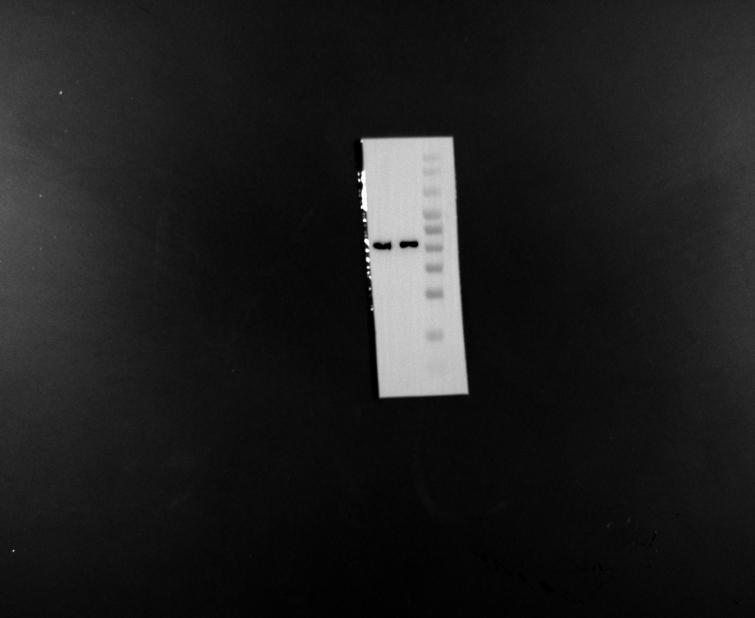
**

**Figure4 I**

POU2F1

**
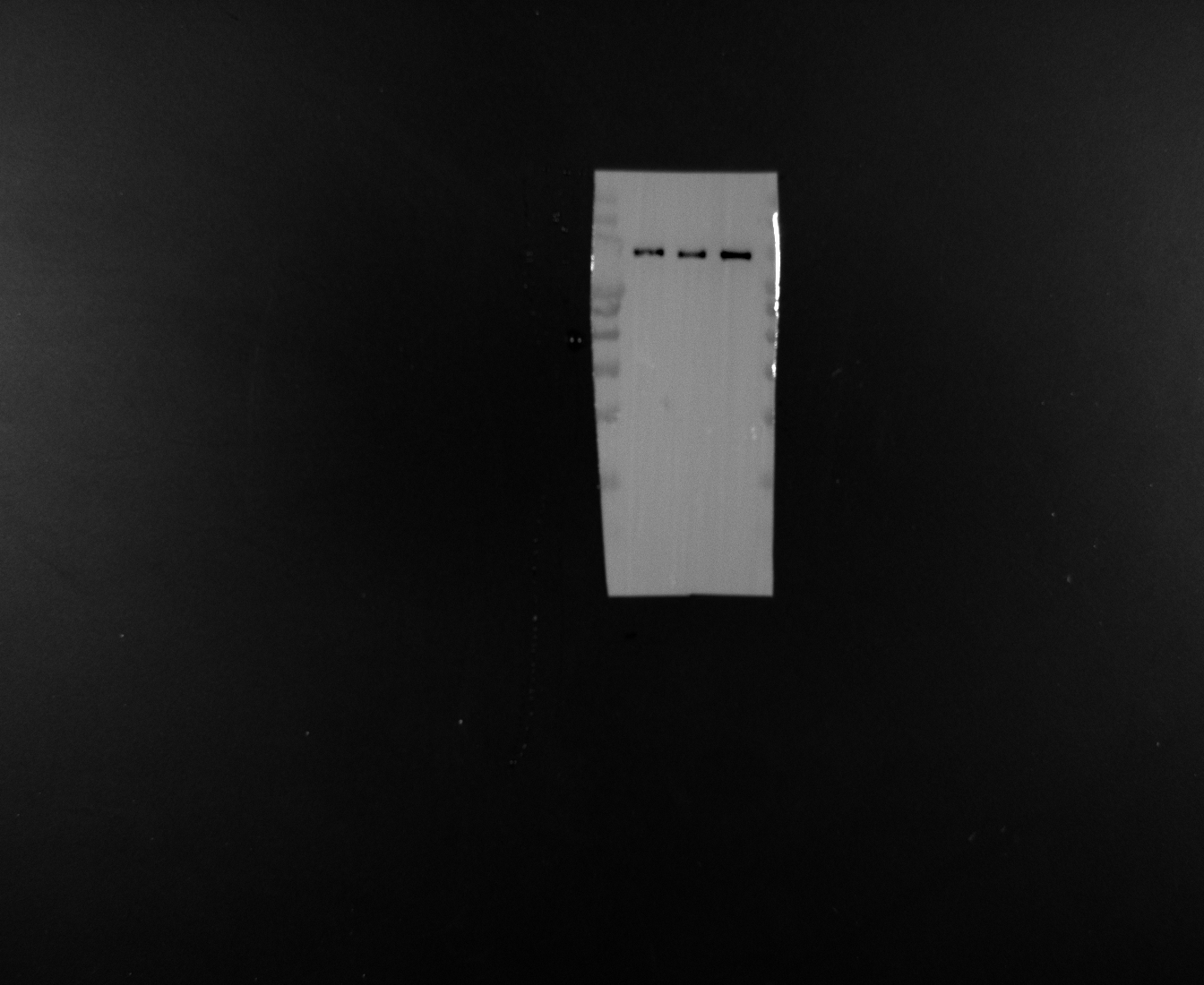
**

β-actin

**
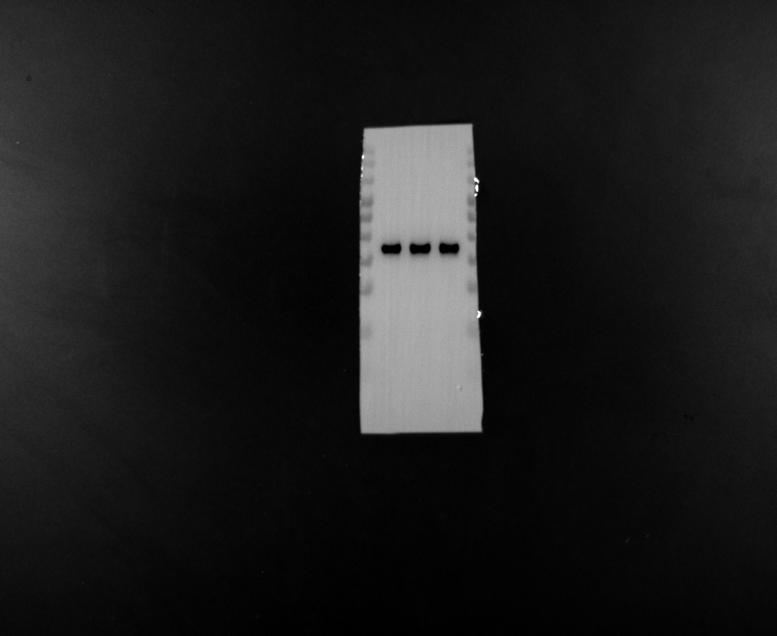
**

**Figure5 D**

HK2

**
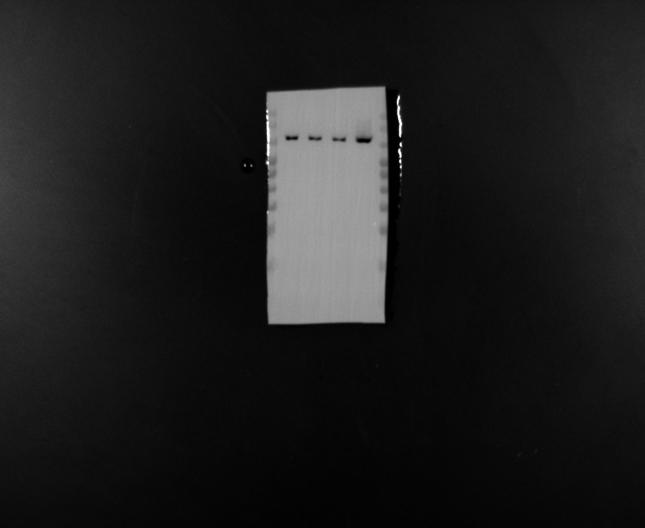
**

PGK1

**
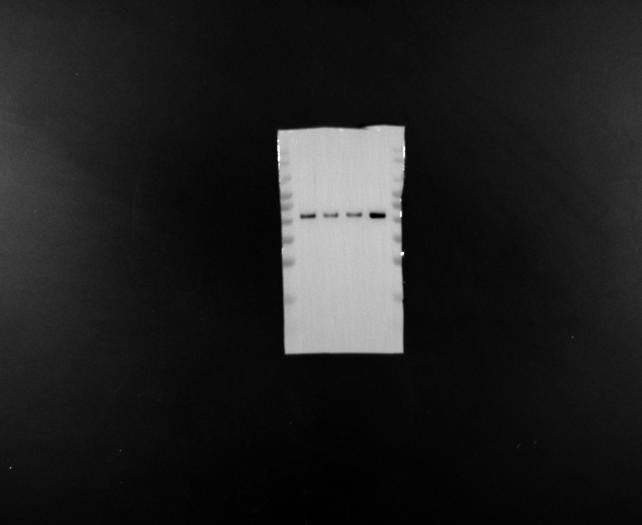
**

PKM2

**
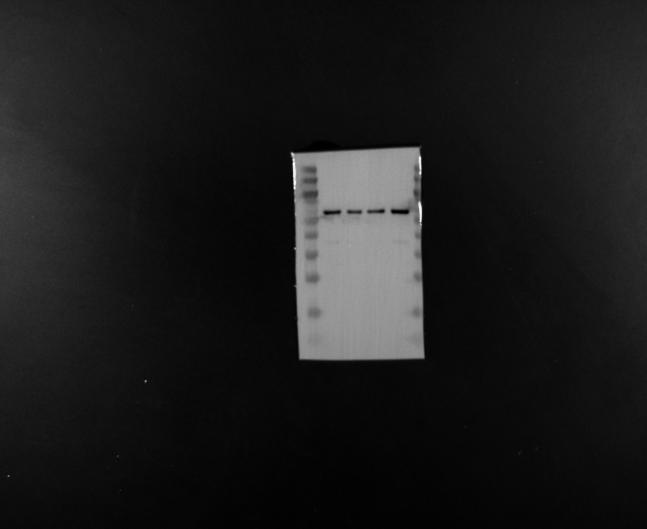
**

β-actin


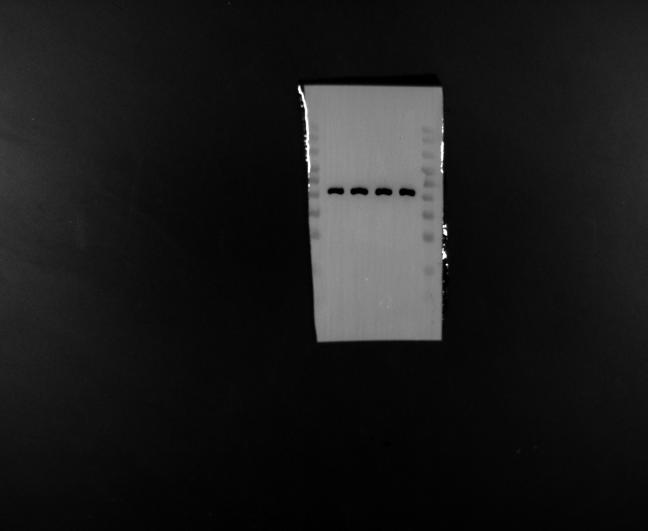


**Figure6 A**

POU2F1


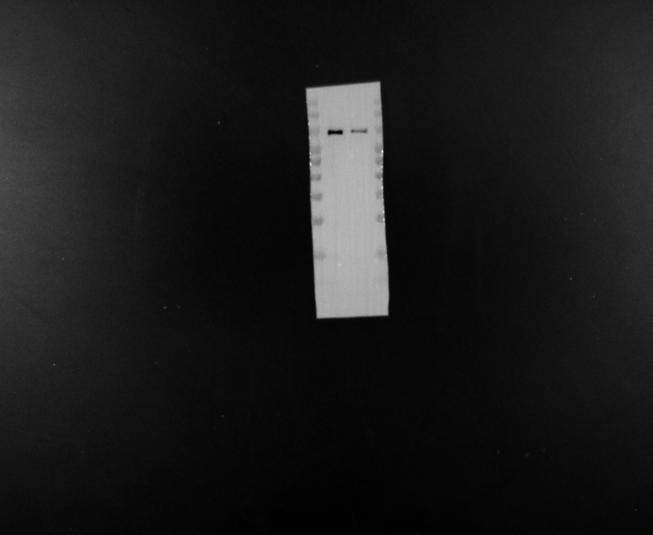


β-actin


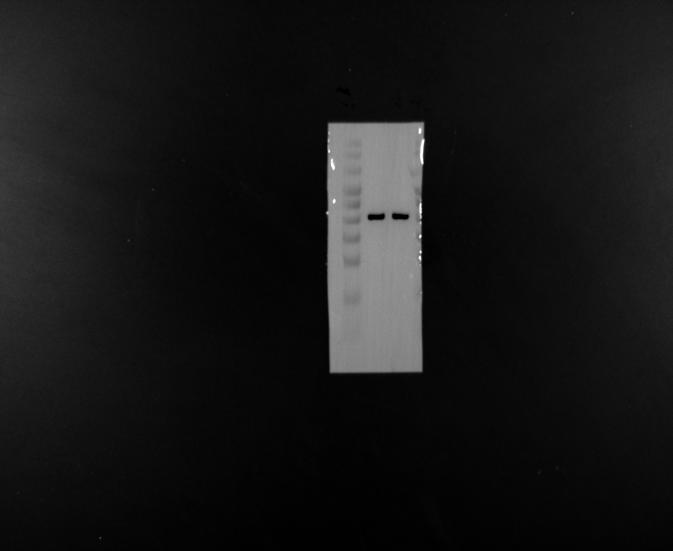


**Figure6 E**

Bax


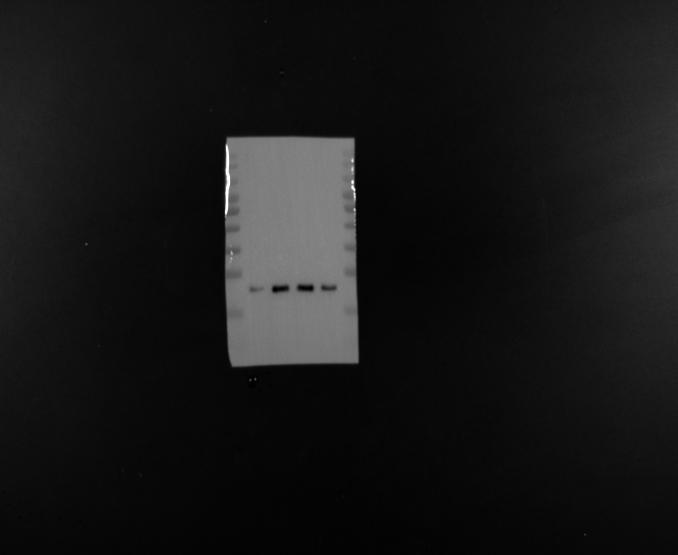


BCL-2


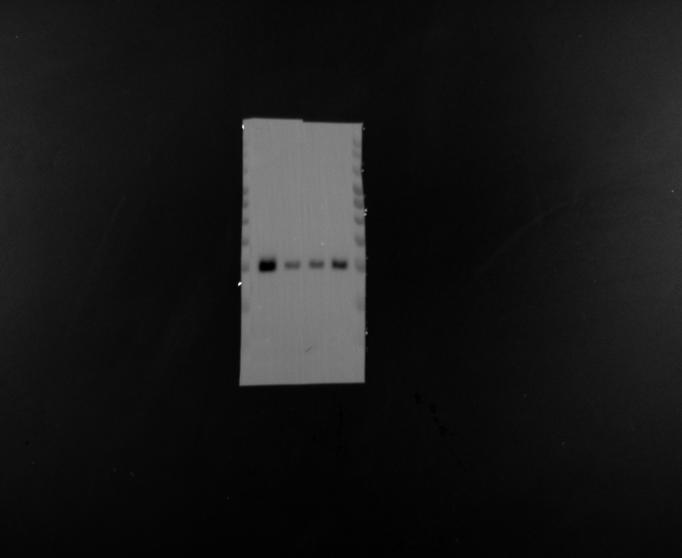


Casepase-3


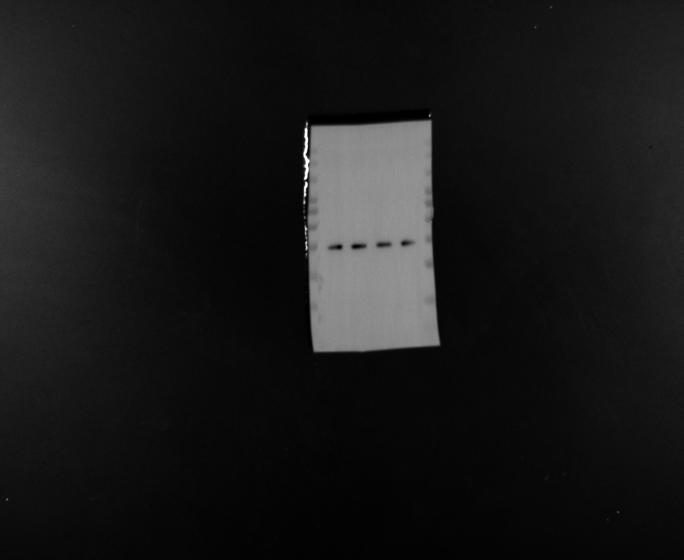


Cleaved casepase-3


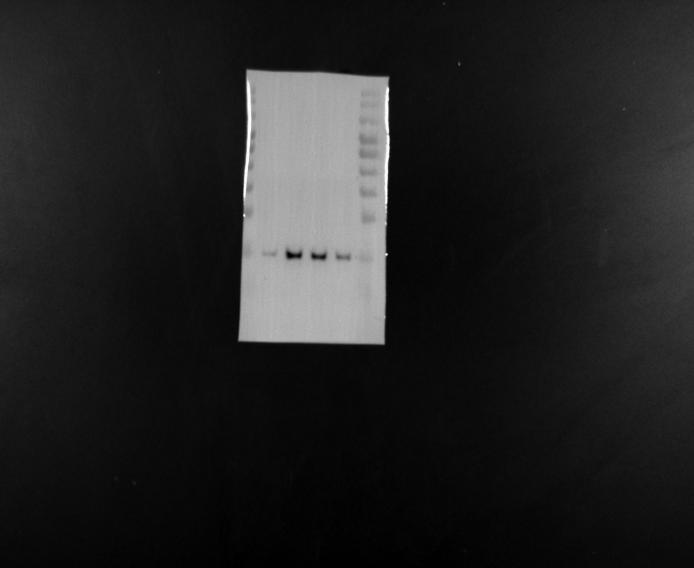


β-actin


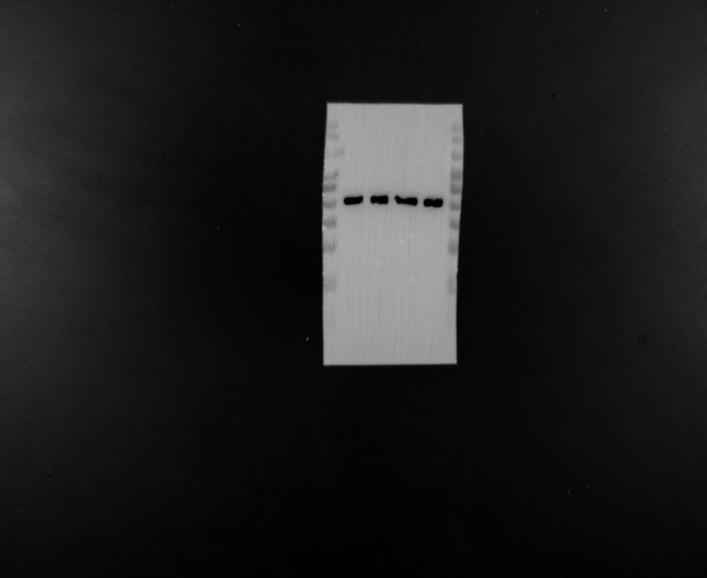


**Figure7**

HK2


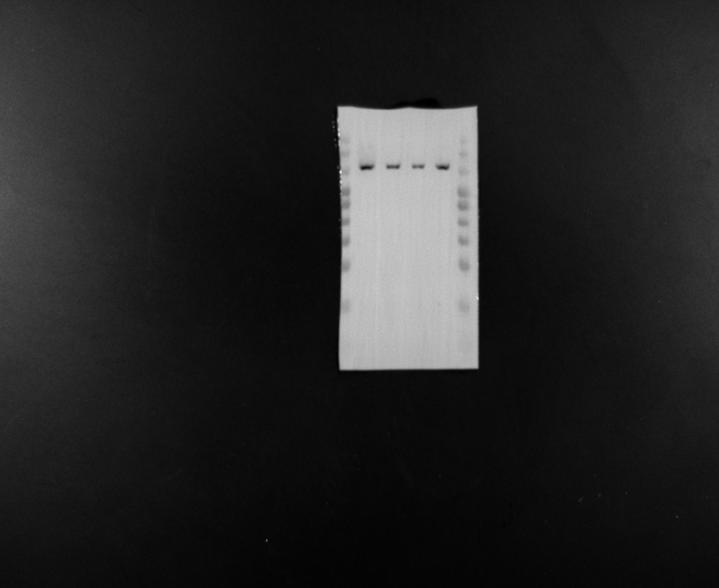


PGK1


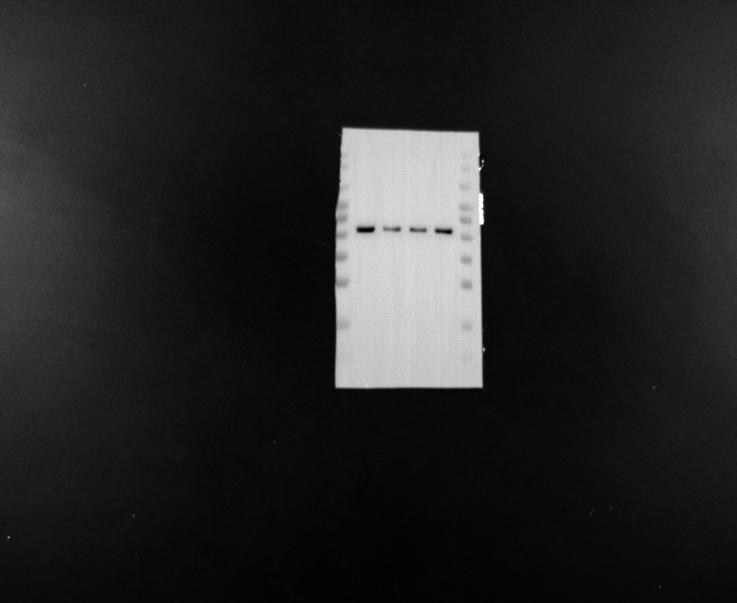


PKM2


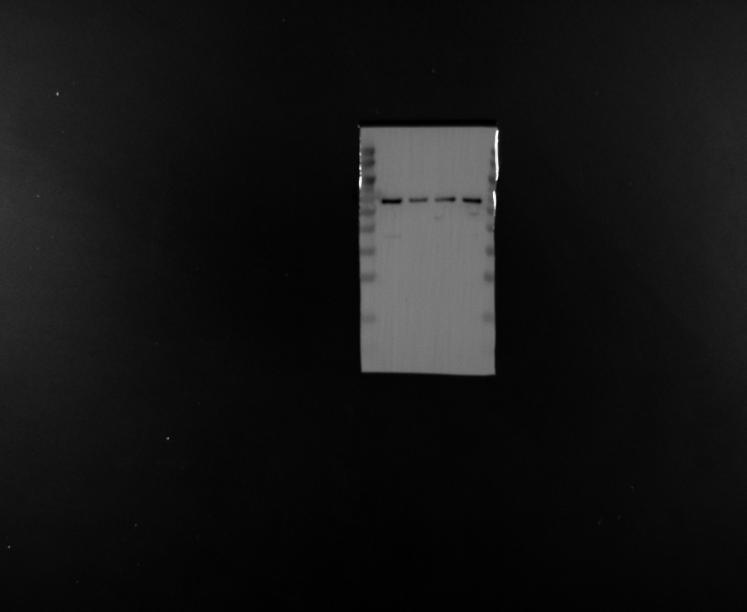


β-actin


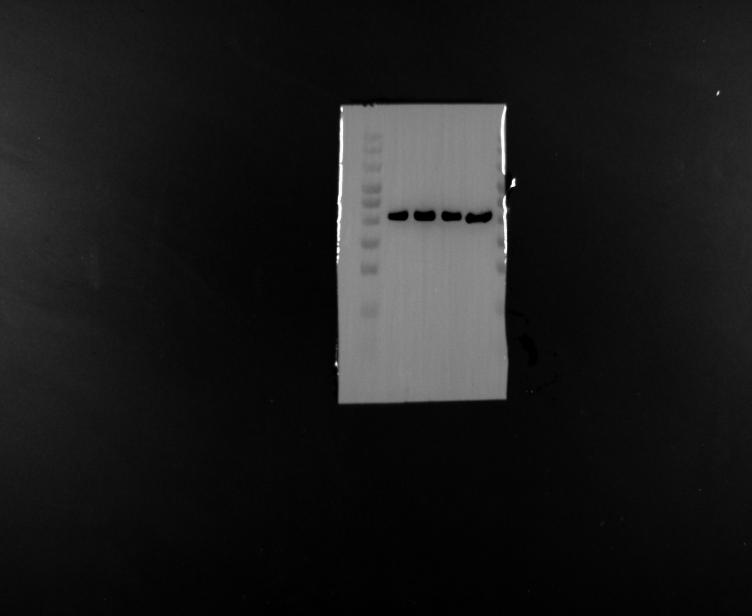

Supplement: Supplementary file 1 — Supplementary Material 1 [file 40659_2026_678_MOESM1_ESM.docx]
